# Effects of different arterial occlusion pressures during blood flow restriction exercise on muscle damage: a single-blind randomized controlled trial

**DOI:** 10.1038/s41598-025-11654-y

**Published:** 2025-07-31

**Authors:** Kevin Happ, Sarah Barawi, Daniel Niederer, Carsten Schwiete, Christine Heinrich, Alexander Franz, Patrick Wahl, Michael Behringer

**Affiliations:** 1 Department of Sports Sciences, Goethe University Frankfurt, Ginnheimer Landstraße 39, 60487 Frankfurt Am Main, Germany; 2 Institute of Occupational, Social and Environmental Medicine, Goethe University Frankfurt, Frankfurt/Main, Germany; 3 Department of Orthopedics and Trauma Surgery, University Hospital Bonn, Bonn, Germany; 4 Department of Trauma Surgery and Orthopedics, BG Klinik Ludwigshafen, Ludwigshafen, Germany; 5 Section Exercise Physiology, German Sport University Cologne, Cologne, Germany; 6 The German Research Centre for Elite Sport Cologne, German Sport University Cologne, Cologne, Germany

**Keywords:** Blood flow restriction exercise, Arterial occlusion pressure, Exercise-induced muscle damage, Biomarkers, Diagnostic markers, Predictive markers, Rehabilitation, Biomarkers, Cell death

## Abstract

Blood flow restriction (BFR) training has been shown to induce exercise-induced muscle damage (EIMD) in some cases, although findings are inconsistent and the influence of the applied arterial occlusion pressure (AOP) remains unclear. This single-blind, randomized controlled trial investigated the effects of different percentages of AOP on EIMD and acute physiological responses in 40 participants allocated to four groups: no pressure (NP), low pressure (LP; 50% AOP), medium pressure (MP; 75% AOP), and high pressure (HP; 100% AOP). Participants performed unilateral knee extensions at 30% of their one-repetition maximum up to four sets of 20 repetitions or until failure. EIMD was primarily assessed by the changes in isokinetic peak torque 24 h, 48 h and 72 h post-exercise (Δ to baseline). Secondary markers included perceived pain, blood biomarkers (creatine kinase, myoglobin) and muscle swelling. Additionally, acute physiological responses were assessed, including continuous measurement of muscle oxygen saturation (SmO_2_) during exercise, perceived exertion (RPE) immediately after the exercise bout, and blood lactate concentration measured at 1, 3, 7, and 10 min post-exercise. NP showed greater strength loss at 24 h post-exercise compared to MP (MD = − 9.95, *p* = .042, 95% CI [− 19.7, − 0.19]) and HP (MD = − 10.51, *p* = .034, 95% CI [− 20.52, − 0.49]). Pain ratings were higher in NP compared to MP (*p* = .001) and HP (*p* = .003) at 24 h post, and remained elevated at 48 h compared to MP (*p* = .003) and HP (*p* = .047). NP and LP completed more repetitions than MP and HP. HP exhibited a greater reduction in SmO_2_compared to NP. Perceived exertion was higher in MP and HP. LP showed higher average lactate concentrations than NP (*p* = .020). CK and MB responses showed no time-specific group differences. These findings suggest that BFR training, even at higher pressures, does not increase EIMD compared to free-flow exercise, and that MP and HP may even attenuate strength loss and pain following exercise.

**Supplementary Information:**

The online version contains supplementary material available at 10.1038/s41598-025-11654-y.

## Introduction

Blood flow restriction training (BFR) combines low-load resistance exercises with special cuffs, applied to the exercising extremities, that partially restricts blood flow to the working muscles. This training typically utilizes loads between 20 and 50% of one-repetition maximum (1RM), effectively promoting muscle growth comparable to high-load resistance training while eliciting moderate increases in strength^1–5^. Today, low-load BFR training is recognized as a valuable tool in various fitness and therapeutic programs. It is embraced by athletes to improve performance, by physiotherapists in pre- and early rehabilitation, when high loads cannot be tolerated, and by the general population for its efficiency in achieving fitness goals with reduced mechanical loads and joint stress^6,7^.

Despite these benefits, concerns about safety and the potential for exercise-induced muscle damage (EIMD) have persisted ever since this training method was established^6–8^. This is mainly due to the reason that studies investigating the relationship between BFR training and muscle damage show mixed results^3,6,7,9^. Some studies report increased creatine kinase (CK) and myoglobin (MB) concentrations^10–12^, edema^10,12,13^, delayed onset muscle soreness (DOMS)^10,12,14^, prolonged strength decrements^10,12,13,15^, signs of damage at the fiber level^15,16^, and even cases of rhabdomyolysis^17–19^ after acute bouts with BFR. However, on the other hand, there is considerable evidence that BFR results in minimal or no muscle damage at all, with only minor strength impairments observed up to 24, at most 48 h after training^3,7–9^.

The reason why BFR training could potentially lead to increased EIMD is nevertheless plausible and is due to the fact that metabolic stress can trigger numerous interlinked mechanisms that ultimately lead to muscle cell damage or increase susceptibility to mechanically induced disruption by reducing muscle fiber resilience^20^. During low-load (LL) exercise, muscle fibers are stressed by repetitive contractions against light external resistance over a prolonged period of time, resulting in decreased ATP availability, a drop in pH and the accumulation of metabolic by-products such as lactate^21,22^. This metabolic stress activates several interrelated mechanisms that contribute to muscle damage. A key factor is the loss of calcium homeostasis caused by impaired calcium pumps due to ATP depletion, leading to an influx of excess intracellular calcium^20,21^. This calcium overload triggers proteolytic and phospholipolytic activation, which damages myofibrillar proteins and disrupts membrane integrity^6,8^. At the same time, the increased calcium concentration impairs the function of the mitochondria^23^, which leads to increased production of reactive oxygen species (ROS)^23,24^. These ROS cause oxidative damage to lipids and proteins, further destabilizing the cell membrane and impairing cell structures. Moreover, ischemia promotes anaerobic metabolism, resulting in the accumulation of metabolites such as lactate. As a consequence, metabolic acidosis occurs, which can affect enzyme activity and protein structure, leading to muscle fatigue and damage^6,8,9^. Thus, the combined mechanisms of metabolic stress-induced physiological responses and repeated low-resistance contractions lead to a temporary loss of their membrane integrity and structural changes eventually resulting in muscle fiber damage^21^.

One parameter of BFR training that regulates these responses and varies greatly between studies is the arterial occlusion pressure (AOP) applied, expressed as a percentage of full arterial occlusion. In the context of BFR training, higher percentages of AOP induce greater ischemia and faster metabolite accumulation^25^. However, assuming equal load, increasing the pressure reduces the total mechanical work that can be performed (defined as repetitions x load) due to accelerated fatigue^26^. Thus, training with a high level of AOP may increase metabolic stress but reduce total mechanical work. In contrast, lowering the applied AOP would reduce metabolic stress while allowing for greater mechanical work until fatigue. Yet, we know that total mechanical work through accumulative low resistance exercise itself also has a considerable impact on muscle damage^27–29^.

Hence, the current understanding is that both metabolic stress and total mechanical work have an influence on the extend of EIMD^30–32^. Yet, the relationship between the two contributors will be inversely affected by the level of AOP during BFR training. Therefore, these two damage mediators behave on a continuum during training close to muscle failure and can only be manipulated in terms of their emphasis by shifts in AOP used. However, no study has investigated the relationship between applied AOP and muscle damage under internal load near muscle failure. Previous research has primarily focused on equivalent external loads, providing valuable insights into mechanical stress mechanisms. Yet, given the interplay between mechanical and metabolic factors in EIMD, this approach captures only a subset of the underlying mechanisms. Based on a reciprocal relationship and the assumption that both factors contribute equally to muscle damage, we hypothesize that the overall extent of EIMD will not differ between different occlusion pressures and non-BFR training, as the opposing effects of metabolic stress and mechanical work are expected to balance each other out.

We chose strength reduction as the primary functional marker as it has been shown to be the best indirect marker of EIMD^33,34^. We also included secondary measures such of CK, MB, subjective pain perception and swelling to capture biochemical, structural and perceptual aspects of EIMD that complement the primary functional assessment. In addition, to accurately assess the specific contributions of metabolic stress and total mechanical workload to EIMD, we also measured lactate accumulation, muscle oxygen saturation (SmO_2_) and rating of perceived exertion (RPE) as indicators of internal load, and mechanical work through repetitions performed and total exercise volume as indicators of external load. In addition to our primary and secondary outcomes, we included exploratory measures such as respiratory exchange ratio, thermographic skin temperature, tensiomyography, and shear wave elastography to further investigate metabolic and structural responses to BFR. These additional variables were used to assess their potential utility in mapping damage or physiological load profiles.

This comprehensive approach aims to clearly identify optimal BFR conditions that maximize training efficiency while minimizing risk by providing practical guidance for safe BFR training practices while providing insight into the underlying mechanisms of EIMD under BFR conditions. This study aims to fill this gap by investigating how different percentages of AOP affect the extent of EIMD compared to free flow training.

## Methods

### Experimental approach

The present study was a single-blind randomized controlled trial (RCT) using a parallel design with four groups. The experiment complied with the standards set by the Declaration of Helsinki and was approved by the Local Ethics Committee of the Department of Psychology and Sports Sciences at Goethe University Frankfurt (Lokale Ethikkommission FB05, Goethe-Universität Frankfurt am Main) under the reference number 2023-08. The ethics vote is not publicly accessible but is available upon request. The study has been registered in the German Clinical Trials Register (DRKS) under the ID DRKS00032247 at the 12/07/2023.

### Subjects

Eligibility criteria for participants were: age 18–35 years and recreationally active, defined as engaging in moderate- to high-intensity physical activity at least twice per week, without the requirement to participate in a specific sport or structured resistance training. Exclusion criteria were: delayed onset muscle soreness at inclusion, injuries, acute or chronic illnesses, taking medication, previous damage to the venous system (e.g. varicose veins, vascular surgery, extremities with dialysis access), high blood pressure, circulatory and coagulation disorders (e.g. condition after transient ischemic attack, stroke, angina pectoris, myocardial infarction, peripheral occlusive disease).

Participants were instructed to avoid strenuous or unaccustomed physical activity during the study period. They were informed about the purpose of the study, but no information was given about which group they were assigned to. These measures were taken to minimize both psychological and physical factors that could influence muscle soreness independent of the intervention. In addition, they were informed about the experimental risks and signed a consent form prior to the examination.

### Procedures

One week prior to the intervention, participants performed a single-leg strength test to determine their one-repetition maximum (1RM) for full knee extension (90°), at a knee extensor machine (Milon industries GmbH, Germany). The same machine was used for the intervention. In addition, they were familiarized with the isokinetic dynamometer to ensure they were comfortable with the testing procedures and to minimize potential learning effects during peak torque measurements, which were used to assess muscle damage.

**Fig. 1:**
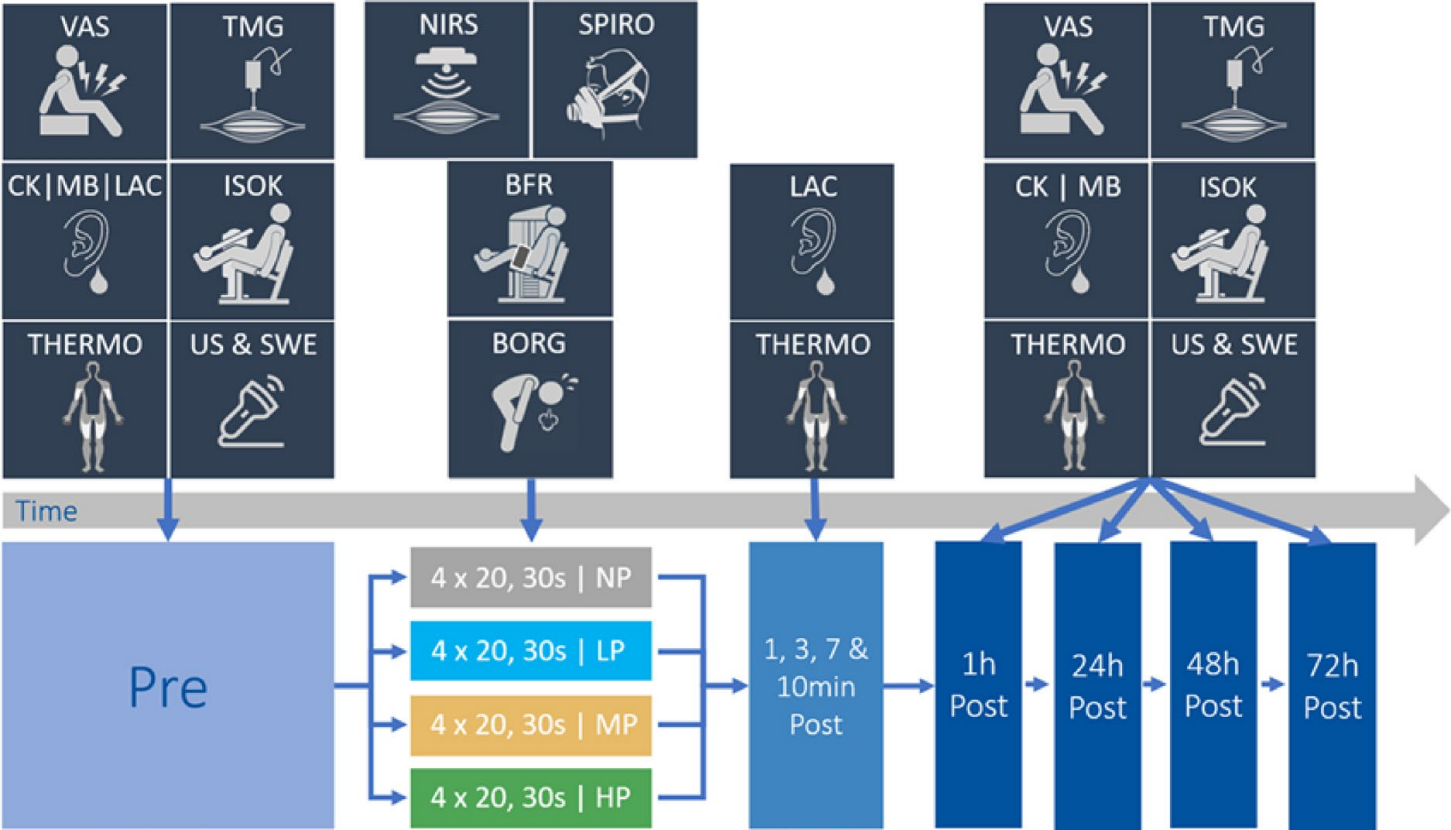
Schematic timeline of the study design including measurement methods and interventions protocols. *VAS* visual analogue scale, *TMG* tensiomyography, *CK* creatine kinase, *MB* myoglobin, *LAC* lactate, *ISOK* isokinetic peak torque test, *thermo* thermography, *US* ultrasound, *SWE* shear-wave elastography, *NIRS* near infrared spectroscopy, *SPIRO* spirometry, *BFR* blood flow restriction, *BORG* rating of perceived exertion, *NP* no pressure, *LP* low pressure (50% of AOP), *MP* medium pressure (75% of AOP), *HP* high pressure (100% of AOP).

At the start of the experiment, participants began with a series of tests to evaluate their individual baseline of muscle damage markers. It consisted of a fixed sequence of diagnostic procedures that were repeated 1 h, 24 h, 48 h and 72 h after the intervention to determine potential changes and muscle damage. The measurements were conducted in the following order: muscle soreness (VAS), capillary blood sampling for CK and Mb, thermography, muscle thickness (ultrasound), muscle stiffness (shear-wave elastography), muscle contractility (tensiomyography), and isokinetic peak torque. The entire experimental protocol and methods used are shown in Fig. 1.

Prior to the intervention, participants sat on the training device and were fitted with near-infrared spectroscopy (NIRS) sensors and the spirometry system. After sensor placement, both systems were calibrated for 10 min under resting conditions to ensure signal stability and to establish individual baselines. The individual AOP was then determined using Delfi’s Personalized Tourniquet System (Delfi Medical Innovation, Canada), which automatically inflates the cuff until the minimum pressure required to stop arterial blood flow is reached, ensuring high reliability^35^. The measurement took place in a seated position with the leg supported horizontally at hip height. Cuffs were placed as proximally as possible on the thigh while maintaining comfort. Delfi’s Easi-Fit cuffs were used, with a cuff width of 11.5 cm and length (46–86 cm) individually selected according to thigh circumference, following the manufacturer’s guidelines. This ensured appropriate pressure distribution and accounted for differences in limb size. In order to avoid any immediate influence from the AOP procedure, participants rested for 5-min before starting the exercise.

The intervention consisted of performing unilateral knee extensions with the dominant leg, which was defined as their kicking leg. Participants were seated with a backrest tilted at 90°, and the seat position was adjusted individually to match their anthropometric characteristics. The knee extension exercise covered a range of motion from 90° to 170° at rest. This setting ensured that the participants were able to extend almost the full range under load without the risk of hyperextension due to compression of the padding. The range of motion was limited with a mechanical lock and was used to consistently determine the number of repetitions completed.

The intervention regimen was identical for all four groups in terms of exercise parameters. Participants completed up to four sets of 20 repetitions each, with 30 s rest between sets, using 30% of their 1RM, or until muscle failure was reached. Muscle failure was defined as the inability to complete full range of motion in three consecutive attempts. If participants were able to regain full range of motion during these three attempts, previous attempts were considered completed repetitions, ensuring that participants never exceeded 20 repetitions per set, including failed trials. During the exercise, participants were instructed to maintain a 2-1-2 s cadence, beginning with the concentric phase, followed by isometric and eccentric. Throughout the exercise, NIRS and spirometry data were recorded continuously. Immediately after completing the exercise bout, the rating of perceived exertion (BORG) was obtained. Capillary blood lactate concentration and thermographic images of the thigh were collected at 1, 3, 7, and 10 min post-intervention.

The only difference between groups was the cuff pressure applied during the intervention. While in the first group the cuff was applied without inflation (No Pressure, NP), in the second group a low pressure of 50% (LP), in the third group a medium pressure of 75% (MP) and in the fourth group a high pressure of 100% (HP) of the individually measured AOP was applied. The cuff was kept inflated until the end of the exercise. The setting of the BFR cuff and the corresponding pressure were applied by an instructed assistant and were unknown to the participant and investigator. Participants were also verbally encouraged during training.

## Measurements

### Primary outcome

#### Isokinetic peak torque

For isokinetic peak torque diagnostics, we used a dynamometer (Isomed 2000, D&R Ferstl GmbH, Germany) with a sampling frequency of 200 Hz to test the knee extensors of the dominant leg. Participants were seated with an 80° hip flexion angle, and the lever arm was adjusted to align the axis of rotation with the knee joint, providing clean and comfortable contact throughout the movement. The range of motion was set between 180° (full knee extension) and 90°. Each repetition started with the eccentric phase, immediately followed by the concentric phase in a continuous motion. Testing speed was set at 60°/s, as recommended for detecting muscle damage-related strength impairments^36^. The peak torque (Nm) of concentric as well as eccentric phase was used for evaluation. Change scores (delta values) relative to baseline were calculated for each phase to assess strength impairments over time.

Prior to baseline testing, participants completed a standardized warm-up. This included one set of 10 repetitions at approximately 50% of their estimated peak concentric torque (based on 1RM), followed by a 3-min rest. They then performed a second ramped set of 6 repetitions: 2 at 50%, 2 at 70%, and 2 at 90% of the anticipated concentric peak torque. Afterward, participants were given the option to perform an additional warm-up set if desired. Following a 5-min rest, participants proceeded to the isokinetic peak torque measurement. The maximal strength testing protocol consisted of a single set of 5 maximal repetitions, designed to minimize additional muscle damage during follow-up assessments. For all subsequent tests, warm-up targets were adjusted based on each participant’s true concentric peak torque established at baseline. The experimenter who supervised the experiment and worked with the participants was blinded to their group allocation.

### Secondary outcomes

#### Delayed onset muscle soreness

The delayed onset of muscle soreness was assessed using a visual analogue scale (VAS). This scale consisted of a 100 mm horizontal line with endpoints labeled “not painful at all” and “most pain imaginable”. During the VAS assessment, participants were asked to mark a point on the line that corresponded to their current level of pain. The distance in millimeters from the left endpoint to the participant’s mark was then measured to determine their pain rating.

#### Biomarker

Capillary blood samples were taken from the fingertips for measuring the indirect muscle damage markers creatine kinase (CK) and myoglobin (Mb). The first drop of blood was removed and subsequent drops were taken for measurement. For this purpose, Lithium heparin microvettes with 200 μl nominal volume were used for blood collection. To evaluate blood markers, the sample was then centrifuged at 4° for 10 min at 3000 rotations per minute (RPM). After separation of the blood plasma, CK concentration was analyzed using the Fujifilm FDC NX500 blood analyzer (Fujifilm, Japan). For Mb concentration, we used the i-CHROMA reader (Boditech Med. inc., South Korea). Since we also evaluated lactate concentrations before and immediately after the intervention (1, 3, 7 and 10 min after), a separate sample was taken and then analyzed with the EKF Biosen C-line system (EKF diagnostic GmbH, Germany).

#### Muscle thickness

We measured muscle thickness of the rectus femoris (RF) and vastus lateralis (VL) using the ultrasound system Siemens ACUSON Redwood (Siemens Healthineers, Germany). For the RF, a marker was placed at 50% of the distance between the anterior inferior iliac spine and the upper edge of the patella. For the VL, 50% of the length of the greater trochanter and the lateral epicondyle of the femur was determined and marked for subsequent measurements. Additional measuring points were marked at ± 25 mm from these points for later electrode placement and shear wave elastography. These markings were maintained throughout the study to reduce variability from measurement errors or positional shifts. Muscle thickness was measured in 2D-B-mode (brightness mode) using a 10L4 linear array probe (50 mm wide) with frequency of 3.3–8.9 MHz under minimal pressure in the transverse plane. The frequency was set to “harmonics high” with a steer of 0° (− 2/0/2) to improve image quality by reducing noise and artifacts. Participants positioned themselves on an examination bench and placed their leg on a 135-degree cushion to ensure that the leg was stable and relaxed. Muscle thickness was measured directly on the stored ultrasound images using the in-built measuring tool of the Siemens ACUSON Redwood system from the lower edge of the superior fascia to the superior edge of the near-bone fascia, as described by Jenkins et al.^37^. Measurements were accurate to the millimeter and were taken three times, with the average value used as the final result. The average of the three measurements was subsequently calculated using Microsoft Excel (Microsoft Corp., USA). After each measurement, ultrasound images were stored and used as a benchmark for subsequent measurements to improve between-day repeatability. The experimenter who carried out the measurement was blinded with regards to group allocation.

#### Muscle oxygen saturation

During training, muscle oxygen saturation (SmO_2_) of the rectus femoris muscle was measured using the IDIAG Moxy (Idiag GmbH) near-field infrared spectroscopy (NIRS). After calibration, NIRS was applied approximately 10 min before the intervention and participants were instructed to remain relaxed to avoid influencing the measurement.

The NIRS sensor was placed at the previously described mark on the rectus femoris and secured with a sleeve fitted to the participant’s thigh to prevent slipping and light interference. The start and end of each set were marked throughout the training session. SmO_2_ values were derived by averaging the final 5 s preceding the start mark and the final 5 s following the end mark of the intervention. SmO_2_ was analyzed as the change (Δ) from baseline (pre-intervention) to the end of each individual intervention session. The SmO_2_ levels recorded were analyzed to assess muscle oxygen saturation changes throughout the intervention.

#### Perceived exertion

Immediately after completing the entire intervention session, participants reported their overall perceived exertion using the Borg scale. They provided a single rating that reflected the total effort of the full session, using the 6 to 20 scale, where 7 represented “extremely easy” and 19 represented “extremely exhausting”.

#### Supplementary measures of muscle response to BFR

In addition to the primary and key secondary outcomes, several supplementary parameters were collected to explore broader aspects of the physiological response to BFR training. Respiratory gas exchange (RER) was included to provide insights into the metabolic demands and anaerobic contribution during exercise. Measures such as shear-wave elastography (SWE), thermographic skin temperature, and tensiomyography (TMG) were intended to examine whether they reflect patterns of muscle damage over time. As these variables were not essential for addressing the primary study objectives, detailed methodologies, analyses, and results are presented in the supplementary material.

#### Statistical analyses

An a priori power analysis was conducted using G*Power 3.1.9.7 to determine the required sample size for an ANOVA (fixed effects, omnibus, one-way) with an effect size of f = 0.58 (partial η^2^ = 0.25), α = 0.05, power (1–β) = 0.80, and 4 groups. The effect size was based on the target variable (strength reduction) and derived from published data reporting large between-group and interaction effects on post-exercise MVC loss ^13,38^. To avoid overestimation, the effect size was conservatively adjusted downward. The analysis showed that a total sample size of 40 participants, equating to 10 participants per group, was required to achieve the desired power. Participants were block-randomized into four different groups of n = 10 each, using a computer-based system (www.sealedenvelope.com). The block size was eight, i.e. the participants were assigned in a balanced way. A total of 41 participants were recruited; however, one participant withdrew due to knee pain. The study phases, including the number of participants at each stage, are presented in the schematic flowchart in the supplementary material (SI Fig. 1).

To evaluate the effects of the applied AOP on all primary (concentric and eccentric peak torque reduction (Δ to baseline)) and secondary (pain perception, biomarkers, muscle swelling (Δ to baseline), lactate) outcome variables with repeated measures, linear mixed models (LMMs) were employed. Each LMM included group and time as fixed effects, with their interaction (group × time) modeled to examine differential changes over time. Participant ID was included as a random intercept to account for inter-individual variability in baseline levels and to model the repeated measures structure of the data. Model diagnostics included reporting of marginal R^2^ (variance explained by fixed effects), conditional R^2^ (variance explained by fixed and random effects), and the intra-class correlation coefficient (ICC) to quantify the proportion of variance attributable to individual differences. Outcomes assessed only once (repetitions, RPE) or as single difference values (SmO_2_ (Δ to baseline)) were analyzed using one-way ANOVAs to evaluate group differences. Post-hoc pairwise comparisons were conducted for significant main effects and interactions. These were corrected for multiple comparisons using Bonferroni-Holm adjustments. All continuous variables were checked for normality and homoscedasticity; model assumptions were verified using Statistical significance was set at 5% alpha error. All analyses were performed using IBM SPSS Statistics (Version 27.0).

Finally, we also categorized the degree of EIMD descriptively, following Paulsen’s criteria^36^. This classification was based on the percentage loss of strength over the 72-h period: Mild damage was defined as a decrease in strength-generating capacity of no more than 20% within the first 24 h, with full recovery within 48 h. Moderate damage corresponded to a 20–50% strength loss, with recovery occurring between 48 h and 7 days. Severe damage was characterized by a strength loss exceeding 50% and/or a recovery period extending beyond one week.

## Results

### Participants

All 41 participants initially recruited were randomized and assigned equally to the respective groups. However, one participant from the NP group has been excluded from the post-measurements due to knee pain. Thus, the analysis included 40 measured participants (13 men and 27 women). The baseline characteristics of the total sample size and each group are presented in Table 1.

**Table 1:** Descriptive data of participants.

Parameter	NP (n = 10)	LP (n = 10)	MP (n = 10)	HP (n = 10)	Total (n = 40)	*p*-value
Gender (f|m)	8 | 2	6 | 4	8 | 2	5 | 5	27 | 13	
Age (y)	25.5 ± 3.5	24.3 ± 2.3	25.4 ± 3.6	24.8 ± 2.6	25.4 ± 2.97	0.799
Height (cm)	169 ± 8.38	171 ± 8.20	174 ± 9.94	177 ± 8.37	172.2 ± 8.96	0.202
Weight (kg)	62.6 ± 12.6	69.7 ± 16.1	66.7 ± 11.3	73.1 ± 11.1	68 ± 13	0.326
AOP (mmHg)	212 ± 11.1	237 ± 35	224 ± 24.5	229 ± 26.4	225 ± 26.4	0.186
1RM (kg)	47.3 ± 13.17	51.6 ± 13.91	53 ± 10.15	57.2 ± 10.87	52.2 ± 12.19	0.349
ConPT (Nm)	176 ± 65.10	203 ± 66.47	187 ± 43.80	206 ± 58.02	193 ± 57.72	0.671
EccPT (Nm)	230 ± 56.81	244 ± 67.60	243 ± 52.17	276 ± 73.35	248.2 ± 60.99	0.447
L (mmol/L)	1.0 ± 0.272	1.3 ± 0.548	0.9 ± 0.247	1.1 ± 0.469	1.1 ± 0.412	0.239
SmO_2_	77.44 ± 5.44	71.79 ± 6.19	62.03 ± 6.51	75.71 ± 4.62	71.74 ± 18.2	0.234
CK (U/L)	154 ± 87.60	201 ± 142.59	132 ± 29.03	458 ± 379.7	241 ± 237	0.059
MB (ng/ml)	17.33 ± 10.67	16.2 ± 6.42	18.56 ± 7.35	26.53 ± 25.0	22.27 ± 16.35	0.350
RF TH (cm)	24.4 ± 2.71	26 ± 4.76	24.9 ± 2.53	26.4 ± 4.16	25.4 ± 3.68	0.592
VL TH (cm)	22.5 ± 2.60	25.1 ± 5.42	25.9 ± 4.56	25.5 ± 4.10	24.8 ± 4.36	0.326

The data presented are in means ± SD; Gender distribution differed slightly across groups. Although a Chi-Square test was significant (*p* < .001), expected frequencies in over 50% of cells were below 5, violating test assumptions. The result should be interpreted with caution.; *NP* no pressure; *LP* low pressure; *MP* medium pressure; *HP* high pressure; *AOP* arterial occlusion pressure; *ConPT* concentric peak torque; *EccPT* eccentric peak torque; *L* Lactate; *SmO*_*2*_ muscle oxygen saturation; *CK* creatine kinase; *MB* myoglobin; *RF* rectus femoris; *VL* vastus lateralis; *TH* muscle thickness.

## Muscle damage marker

### Concentric peak torque

**Fig. 2:**
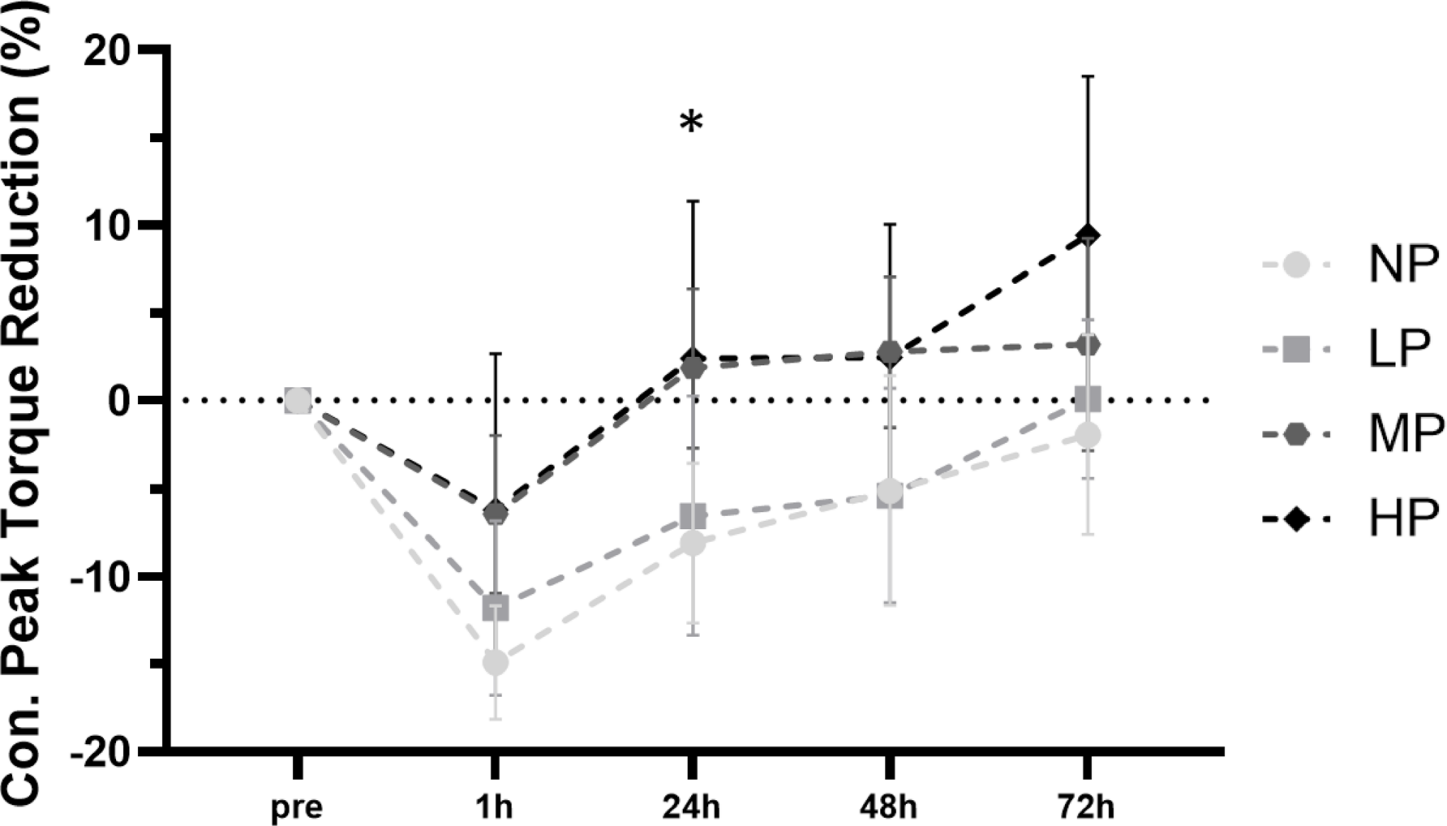
Comparison of exercise-induced concentric peak torque changes from baseline to 1 h, 24 h, 48 h and 72 h post-exercise (mean with 95% CI). *NP* no pressure, *LP* low pressure, *MP* medium pressure, *HP* high pressure, *significant differences between NP and MP & HP of *p* < .05.

Linear mixed model analysis revealed a significant group × time interaction [F (12, 138.28) = 1.82, *p* = 0.050], as well as significant main effects of group [F (3, 36.50) = 4.66, *p* = 0.007] and time [F (4, 138.31) = 26.18, *p* < 0.001] for changes in concentric peak torque following the intervention, indicating group-specific differences in strength impairment over time (Fig. 2). Post-hoc comparisons showed that the NP group experienced greater strength loss at 24 h post-exercise compared to both MP (MD = − 9.95, *p* = 0.042, 95% CI [− 19.7, − 0.19]) and HP (MD = − 10.51, *p* = 0.034, 95% CI [− 20.52, − 0.49]).

The model explained 36.4% of the variance in concentric torque through fixed effects alone (marginal R^2^ = 0.364), and 62.6% when including random effects (conditional R^2^ = 0.626). The intra-class correlation coefficient (ICC = 0.412) indicated that 41.2% of the variance was attributable to between-subject differences, justifying the inclusion of a random intercept for participant ID.

### Eccentric peak torque

**Fig. 3:**
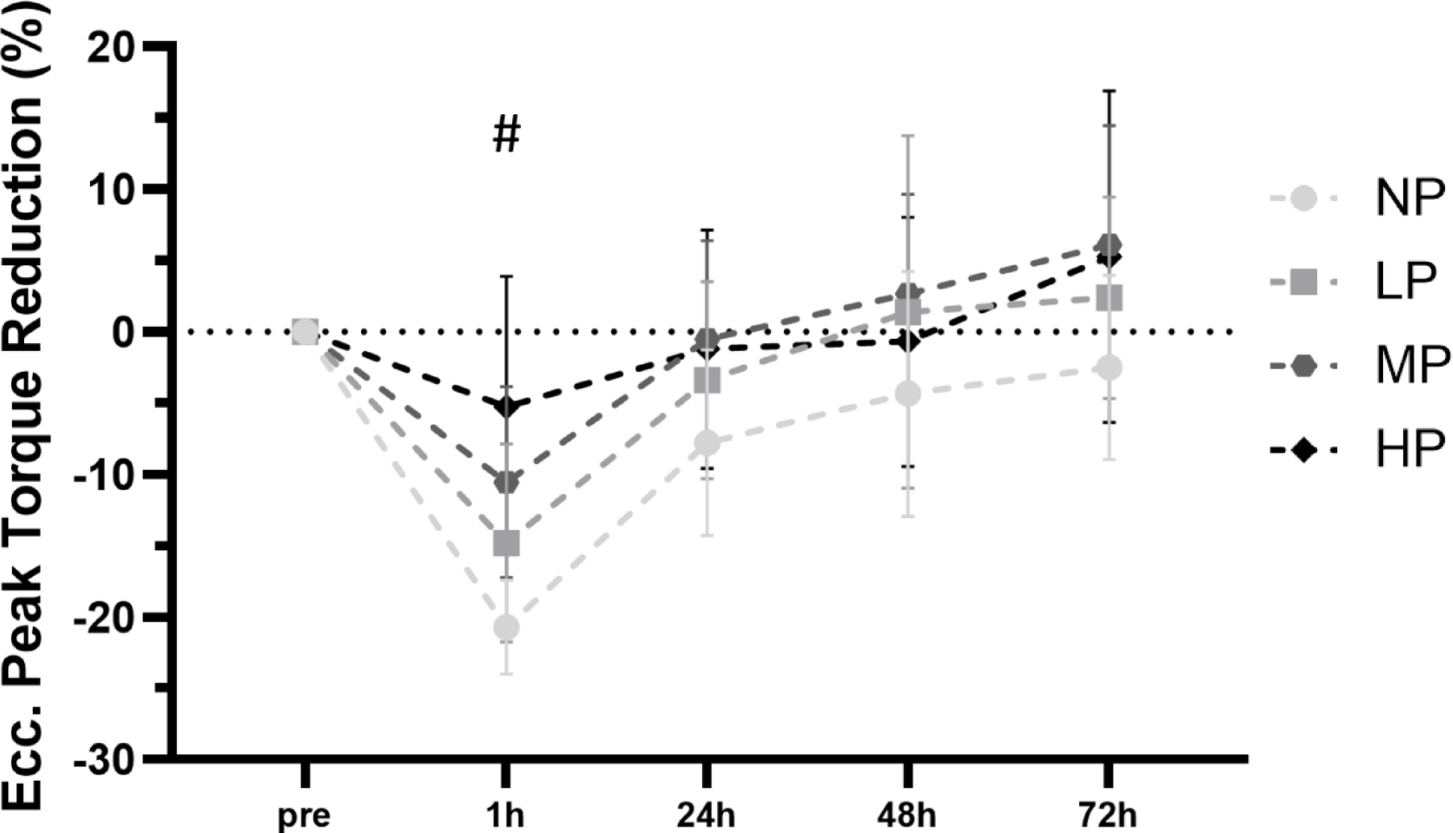
Comparison of exercise-induced eccentric peak torque changes from baseline to 1 h, 24 h, 48 h and 72 h post-exercise (mean with 95% CI). *NP* no pressure, *LP* low pressure, *MP* medium pressure, *HP* high pressure. # significant differences to baseline *p* < .05.

Linear mixed model analysis revealed no significant group × time interaction [F (12, 138.39) = 1.09, *p* = 0.371] and no significant main effect of group [F (3, 36.39) = 2.10, *p* = 0.117], but a significant main effect of time [F (4, 138.42) = 22.75, *p* < 0.001] for changes in eccentric peak torque following the intervention, indicating that strength impairments occurred over time but did not differ between groups (Fig. 3). Post-hoc comparisons for the main effect of time revealed a significant reduction in eccentric peak torque only from pre to 1 h post-exercise (MD = –10.05%, *p* < 0.001, 95% CI [− 14.82, − 5.27]).

The model explained 30.3% of the variance in eccentric torque through fixed effects alone (marginal R^2^ = 0.303), and 54.4% when including random effects (conditional R^2^ = 0.544). The intra-class correlation coefficient (ICC = 0.346) indicated that 34.6% of the variance was attributable to between-subject differences, justifying the inclusion of a random intercept for participant ID.

### Delayed onset muscle soreness

**Fig. 4:**
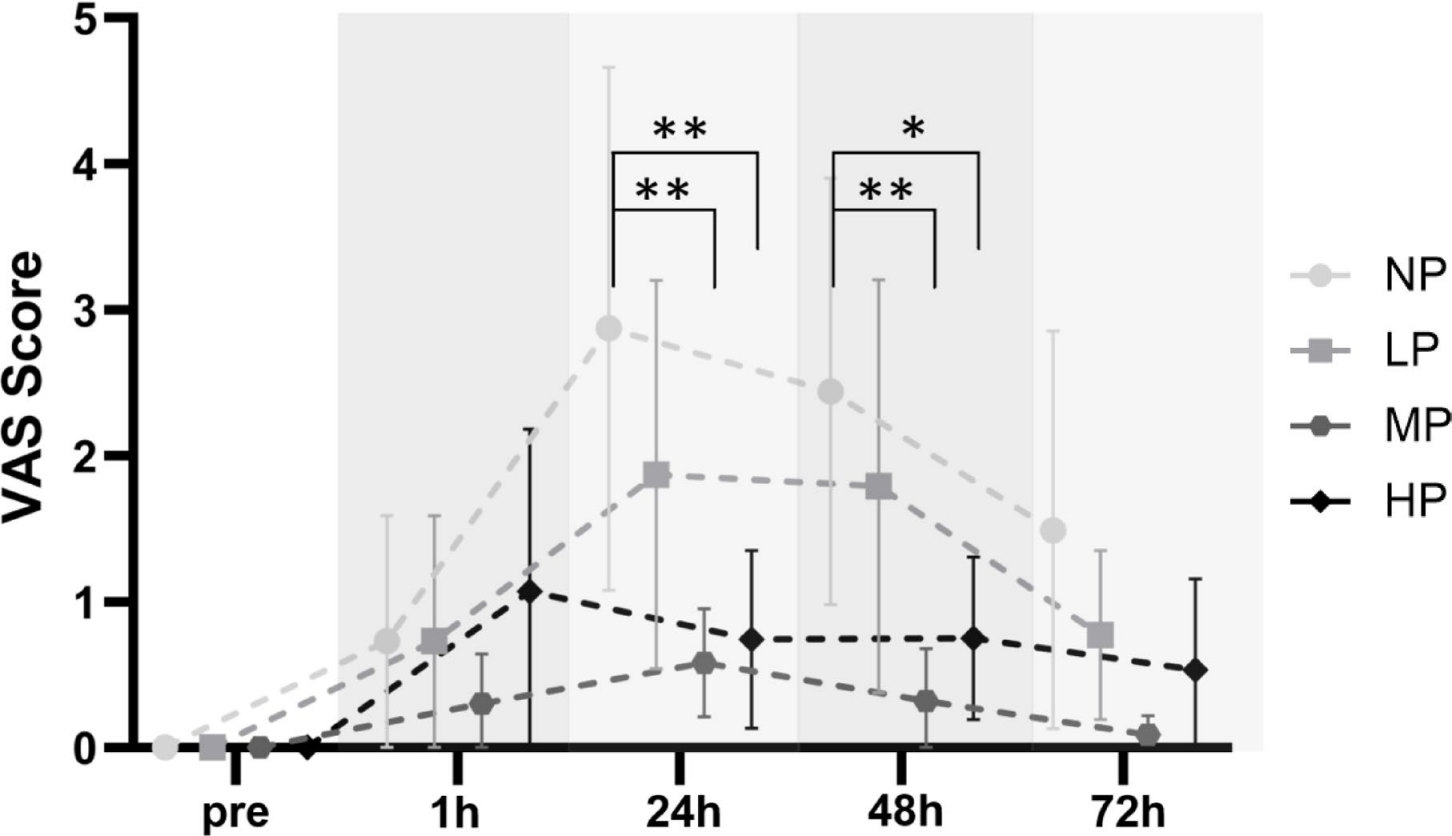
Comparison of exercise-induced muscle soreness changes from baseline to 1 h, 24 h, 48 h and 72 h post-exercise (mean with 95% CI). *VAS* visual analogue scale, *NP* no pressure, *LP* low pressure, *MP* = medium pressure, *HP* high pressure. *significant differences between NP and MP & HP of *p* < .05. **significant difference of *p* < .005.

Linear mixed model analysis revealed a significant group × time interaction [F (12, 142.51) = 2.48, *p* = 0.006], as well as significant main effects of group [F (3, 36.21) = 4.72, *p* = 0.007] and time [F (4, 142.52) = 13.15, *p* < 0.001] for VAS scores following the intervention, indicating group-specific differences in muscle soreness over time (Fig. 4). Post-hoc comparisons showed that the NP group reported significantly greater soreness at 24 h post-exercise compared to both MP (MD = 2.35, *p* = 0.001, 95% CI [0.23, 4.47]) and HP (MD = 2.34, *p* = 0.003, 95% CI [0.22, 4.46]), as well as at 48 h compared to MP (MD = 2.22, *p* = 0.003, 95% CI [0.25, 4.18]) and HP (MD = 1.72, *p* = 0.047, 95% CI [0.30, 3.16]).

The model explained 31.3% of the variance in perceived muscle soreness through fixed effects alone (marginal R^2^ = 0.313), and 49.3% when including random effects (conditional R^2^ = 0.493). The intra-class correlation coefficient (ICC = 0.263) indicated that 26.3% of the variance was attributable to between-subject differences.

## Muscle swelling

### Rectus femoris

Linear mixed model analysis revealed no significant group × time interaction [F (12, 141.79) = 0.80, *p* = 0.646] and no significant main effect of group [F (3, 36.34) = 1.49, *p* = 0.231], but a significant main effect of time [F (4, 141.81) = 96.06, *p* < 0.001] for rectus femoris muscle swelling, indicating a consistent change over time regardless of group assignment (SI Fig. 3). Post-hoc comparisons revealed that RF muscle thickness significantly increased from pre- to 1 h post-exercise (MD = 10.63%, SE = 0.656, T (141.01) = − 16.20, *p* < 0.001), while no significant changes were observed beyond that time point.

These results indicate a transient increase in rectus femoris thickness immediately after exercise across all groups, which returned to baseline within 24 h. Group-specific differences in response were not observed.

The model explained 60.5% of the variance in rectus femoris thickness through fixed effects alone (marginal R^2^ = 0.605), and 70.7% when including random effects (conditional R^2^ = 0.707). The intra-class correlation coefficient (ICC = 0.258) indicated that 25.8% of the variance was attributable to between-subject differences.

### Vastus lateralis

Linear mixed model analysis revealed no significant group × time interaction [F (12, 139.50) = 1.28, *p* = 0.232] and no significant main effect of group [F (3, 36.37) = 2.01, *p* = 0.128], but a significant main effect of time [F (4, 139.53) = 56.97, *p* < 0.001] for vastus lateralis muscle swelling, indicating a consistent change over time regardless of group assignment (SI Fig. 4). Post-hoc comparisons revealed that VL muscle thickness significantly increased from pre- to 1 h post-exercise (MD = 8.65%, SE = 0.693, T (139.42) = –12.48, *p* < 0.001), while no significant changes were observed beyond that time point.

These results indicate a transient increase in vastus lateralis thickness immediately after exercise across all groups, which returned to baseline within 24 h. Group-specific differences in response were not observed.

The model explained 45.8% of the variance in vastus lateralis thickness through fixed effects alone (marginal R^2^ = 0.458), and 67.0% when including random effects (conditional R^2^ = 0.670). The intra-class correlation coefficient (ICC = 0.392) indicated that 39.2% of the variance was attributable to between-subject differences.

## Biomarkers

### Creatine kinase

Linear mixed model analysis revealed no significant group × time interaction [F (9, 96) = 0.66, *p* = 0.735], but significant main effects of time [F (3, 96) = 3.62, *p* = 0.016] and group [F (3, 32) = 3.51, *p* = 0.026] for creatine kinase concentrations following the intervention, indicating overall differences across time and between groups, but without group-specific trajectories (SI Fig. 5). Post-hoc comparisons for time showed that CK concentrations were significantly elevated at 24 h post-exercise compared to baseline (MD = − 78.34 U/L, SE = 26.17, T (96) = –2.99, *p* = 0.021). No other time points differed significantly from pre-values (*p* ≥ 0.070). Although a significant main effect of group was detected, none of the pairwise group comparisons remained significant after correction (*p* ≥ 0.051). Visual inspection suggested that this effect may have been influenced by a small number of participants with elevated CK concentrations throughout the study period in the HP group.

These results indicate a modest, transient increase in CK concentrations 24 h post-exercise across all groups, with no meaningful differences between groups over time.

The model explained 21.6% of the variance in CK levels through fixed effects alone (marginal R^2^ = 0.216), and 85.2% when including random effects (conditional R^2^ = 0.852). The intra-class correlation coefficient (ICC = 0.811) indicated that 81.1% of the variance was attributable to between-subject differences, strongly justifying the inclusion of a random intercept for participant ID.

### Myoglobin

Linear mixed model analysis revealed no significant group × time interaction [F (12, 139.09) = 0.48,  *p* = 0.923] and no significant main effect of time [F (4, 139.09) = 0.91, *p* = 0.455], but a significant main effect of group [F (3, 35.04) = 3.62, *p* = 0.022] for myoglobin concentrations, indicating stable group differences across time. Post-hoc comparisons showed that the HP group exhibited significantly higher overall myoglobin concentrations compared to NP (MD = 19.18 ng/mL, SE = 6.77, T (34.95) = 2.83, *p* = 0.046). No other pairwise group comparisons were statistically significant (*p* ≥ 0.056). Visual inspection suggested that this effect may have been influenced by a small number of participants with elevated MB concentrations throughout the study period in the HP group (SI Fig. 6).

These results suggest that while myoglobin levels did not change significantly over time, overall concentrations were higher in the HP group, potentially reflecting individual variability rather than a consistent group-level effect.

The model explained 17.9% of the variance in myoglobin concentrations through fixed effects alone (marginal R^2^ = 0.179), and 70.0% when including random effects (conditional R^2^ = 0.700). The intra-class correlation coefficient (ICC = 0.635) indicated that 63.5% of the variance was attributable to between-subject differences, justifying the inclusion of a random intercept for participant ID.

## Intervention

### Repetitions

A Welch’s ANOVA showed an effect of applied AOP on repetitions completed [F (3, 15.54) = 53.5, *p* < 0.001, n^2^ = 0.754], indicating different relative mechanical work between groups. Post-hoc comparisons revealed differences between NP and MP (MD = 31.9, *p* < 0.0001, 95% CI [21.18, 42.62]), as well as between NP and HP (MD = 34.90, *p* < 0.0001, 95% CI [25.65, 44.15]). We furthermore found differences between LP and MP (MD = 25.24, *p* = 0.004, 95% CI [8.21, 42.28]), as well as LP and HP (MD = 28.24, *p* = 0.002, 95% CI [11.6, 44.89]).

**Fig. 5:**
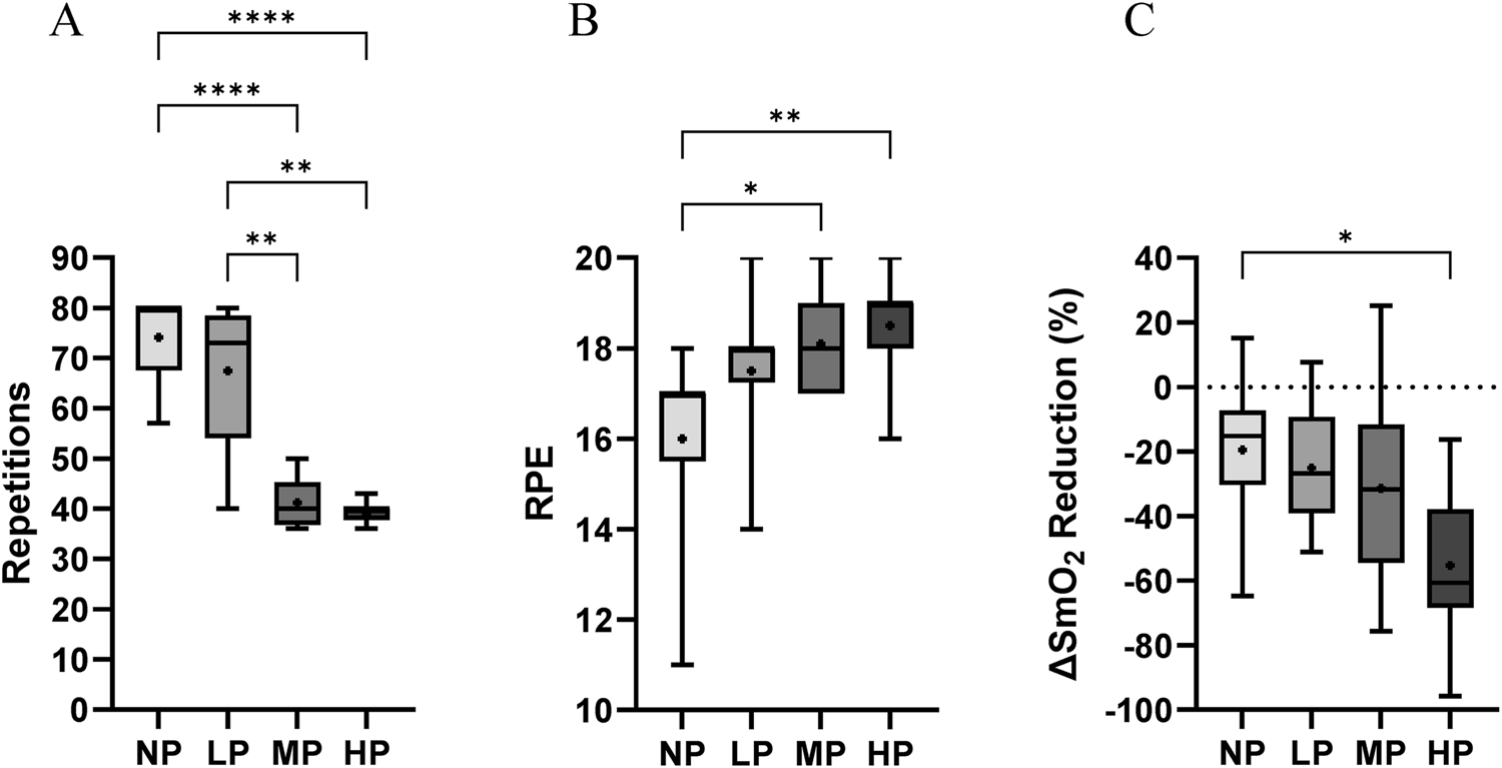
Group differences during intervention. (**A**) Completed repetition during intervention. (**B**) Rate of perceived exhaustion immediately after intervention. (**C**) Changes of oxygen saturation of the m. rectus femoris from baseline to end of intervention. Box and whisker plots show the median (line within the box), the interquartile range (box, spanning Q1 to Q3), and the minimum and maximum values (whiskers). The dot in the middle represents the mean. SmO_2_ = muscle oxygen saturation, *NP* no pressure, *LP* low pressure, *MP* medium pressure, *HP* high pressure. *significant differences of *p* < .05. **significant difference of  *p*< .005. ****significant differences of *p* < .0001.

Therefore, NP (M = 74.1 ± 8.9 SD) and LP groups (M = 67.4 ± 14.8 SD) completed a higher number of repetitions, when compared to the MP (M = 42.2 ± 7.3 SD) and HP group (M = 39.2 ± 1.9 SD) as seen in Fig. 5A.

### Rate of perceived exertion

Results showed an effect of applied AOP on RPE ratings [F (3, 36) = 5.16, *p* = 0.005, n^2^ = 0.301]. The post-hoc test found differences between NP and MP (MD = − 2.10, *p* = 0.02, 95% CI [− 3.94, − 0.26]), and NP and HP (MD = − 2.50, *p* = 0.004, 95% CI [− 4.34, − 0.66]).

These results indicate that participants within MP (M = 18.1 ± 0.9 SD) and HP (M = 18.5 ± 1.0 SD) felt higher effort and subjective perceived exertion than participants within NP (M = 16 ± 2.1 SD) reported once immediately after completion of the entire exercise bout (Fig. 5B).

### Muscle oxygen saturation

Results showed an effect of applied AOP on SmO_2_ reduction (Δ to baseline) at the end of training [F (3, 33) = 3.886, *p* = 0.017, η^2^ = 0.261]. Post-hoc analysis revealed a difference in ΔSmO_2_ only between NP and HP (MD = 35.78%, *p* = 0.016, 95% CI [5.25, 66.32]).

These results show that participants from HP (M = − 55.25 ± 23.66 SD) experienced a greater reduction in SmO_2_ during training compared to NP (M = − 19.47 ± 21.99 SD) (Fig. 5C).

### Lactate

**Fig. 6:**
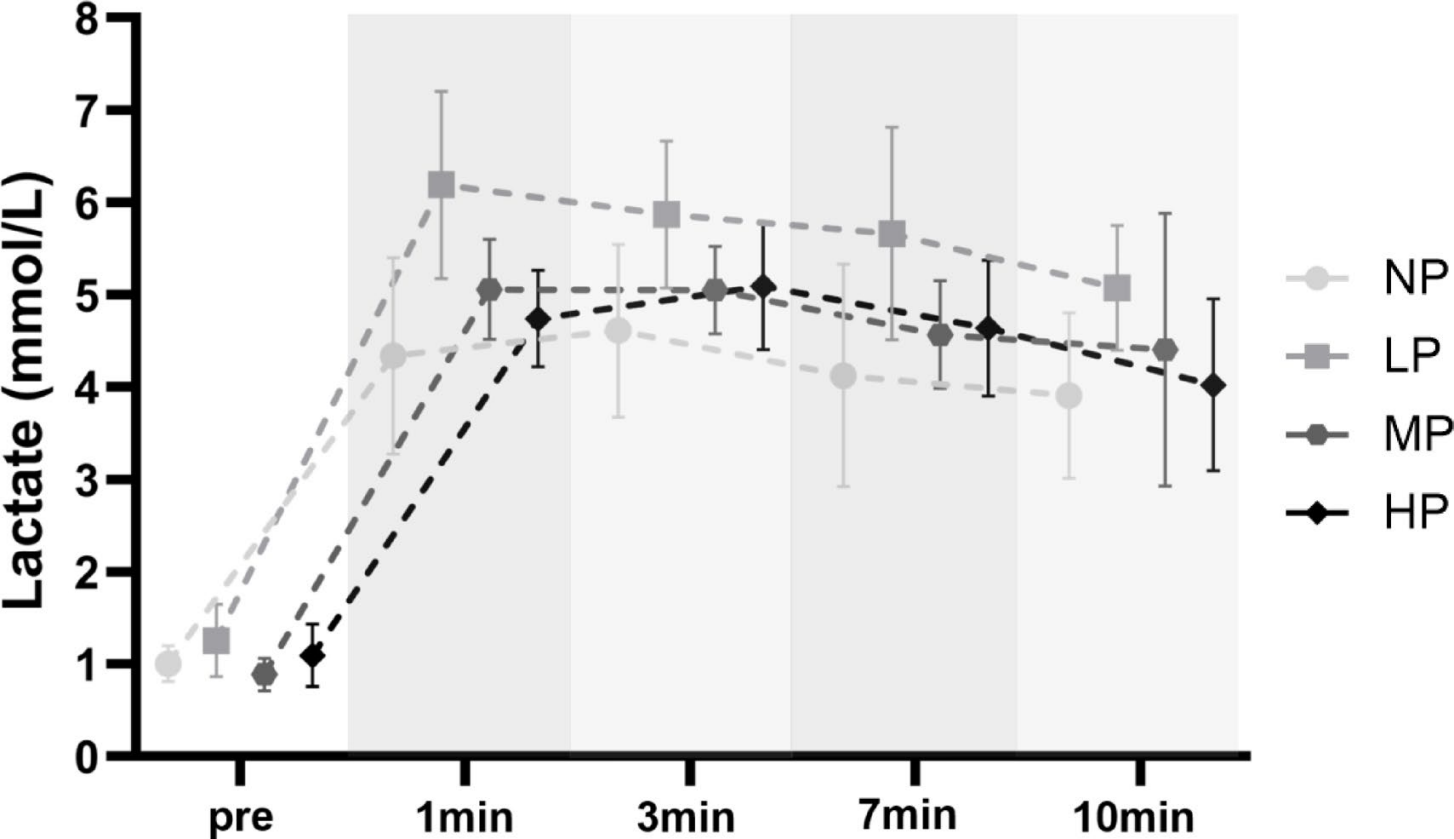
Exercise-induced lactate concentration changes from baseline to 1 min, 3 min, 7 min and 10 min post-exercise (mean with 95% CI). *NP* no pressure, *LP* low pressure, *MP* medium pressure, *HP* high pressure.

Linear mixed model analysis revealed no significant group × time interaction [F (12, 144) = 1.22, *p* = 0.272], but significant main effects of time [F (4, 144) = 183.75, *p* < 0.001] and group [F (3, 36) = 3.87, *p* = 0.017], indicating group differences and a general temporal pattern in lactate responses independent of specific group-time trajectories (Fig. 6). Post-hoc comparisons showed that the NP group had significantly lower overall lactate concentrations compared to LP (MD = − 1.21 mmol/L, SE = 0.38, T (36) = − 3.14, *p* = 0.020). No other pairwise group comparisons reached statistical significance (*p* ≥ 0.062).

These findings indicate that while lactate concentrations varied substantially over time across all groups, participants in the LP group exhibited higher average lactate concentrations than those in the NP condition.

The model explained 67.5% of the variance in lactate concentrations through fixed effects alone (marginal R^2^ = 0.675), and 83.6% when including random effects (conditional R^2^ = 0.836). The intra-class correlation coefficient (ICC = 0.495) indicated that 49.5% of the variance was attributable to between-subject differences, justifying the inclusion of a random intercept for participant ID.

## Discussion

In this study, we investigated the impact of different AOPs during BFR exercise on EIMD. To our knowledge, this is the first study to examine this relationship under internal load conditions near muscle failure, closing an important gap in the literature. While previous studies have primarily focused on external load-matched designs to assess mechanical stress, our approach allowed us to explore the interaction of metabolic and mechanical factors in a setting that better reflects real-world exercise to failure.

To assess this, we focused primarily on strength reduction, widely considered the most valid functional indicator of muscle damage^33,34,36^. The early phase, particularly 24 h and 48 h post-exercise, should be especially valued, as it represents the most sensitive time frame for detecting group differences. This is due to the fast recovery rate after mild to moderate EIMD, which is characterized by a strength reduction of no more than 20% the day after training and recovery to baseline within approximately 48 to 96 h^36^. Although fatigue and EIMD symptoms may overlap, making clear attribution difficult, it is unlikely that the observed group differences in strength reduction were due to prolonged fatigue. While BFR increases fatigability during exercise compared to non-BFR conditions, it does not impair 24-h neuromuscular recovery^39^. This aligns with previous findings showing that key fatigue-related mechanisms, such as motor neuron activity^40^ and metabolic fatigue responses^41^, are comparable between BFR and free-flow exercise. While strength loss was the primary outcome, we also assessed a comprehensive panel of the secondary markers pain perception, CK, MB and muscle swelling of the RF and VL to provide a multi-faceted view of muscle damage. These were evaluated over 1, 24, 48, and 72 h to capture their kinetic progression. Additionally, we categorized the degree of EIMD descriptively, classifying it into mild, moderate, and severe based on strength reduction (Δ1h, 24 h, 48 h, 72 h to baseline) according to the criteria outlined by Paulsen et al.^36^. To complement these findings, data from our supplementary analyses on muscle stiffness (SWE), skin temperature, and muscle contractility (TMG) are presented in SI Figs. 7–9, 10, and 11–14, respectively.

### Muscle damage responses

#### Strength impairments

The major aim of our study was to determine whether BFR training with different AOPs causes greater muscle damage than conventional LL training. Our primary outcome of strength impairments clearly shows that this is not the case. Our results show that the NP group experienced the largest decline in concentric peak torque 24 h after the intervention (mean = –8.1 ± 5.9%), while the LP group showed a comparable reduction. In contrast, the MP (M = 1.9 ± 6.3%) and HP (M = 2.4 ± 11.7%) groups demonstrated no reductions in strength. These findings support previous evidence that BFR exercise does not cause greater muscle damage than LL training when both are performed to muscle failure^38,42^. Even when total workload is matched, strength loss remains comparable between conditions^4,12,15,43–45^, with only one exception^13^. Importantly, this comparison of matched workload reflects a maximal internal load for the BFR group in most cases, whereas the control group operates at a submaximal internal load. Despite this difference, the extent of EIMD does not appear to be greater in the BFR conditions. Considering the frequently discussed role of proximity to failure as a contributing factor to muscle damage^3,6^, it is noteworthy that no enhanced impairment occurred under these adverse circumstances. If anything, there is a trend towards an even smaller reduction in strength capacity with increasing AOP. This may be due to the protective effect of fatigue, which reduces susceptibility to contraction-induced dysfunction, as it may limit overstretching of sarcomeres and thus prevent structural damage^46^.

Interestingly, although reductions in muscle strength are typically most notable in eccentric actions^30^, no differences were found between groups. At 24 h post-exercise, the NP group showed the largest mean decrease in eccentric peak torque (− 7.8 ± 8.5%), with smaller reductions observed in the LP (− 3.4 ± 9.7%), MP (− 0.52 ± 9.6%), and HP (− 1.2 ± 10.8%) groups. Nevertheless, comparable reductions are observed in concentric and isometric movements, which emphasizes that these markers are likewise reliable and valid indicators of EIMD^30,36^. One explanation why we found differences only in concentric contractions may be due to testing specificity, as using the same speed of motion in damage and testing protocols enhances sensitivity^34^. Fatigue during the intervention likely caused less consistent muscle contractions in the eccentric phase. Given that the isokinetic test had 1.5 s for each phase of the movement, the concentric peak torque test therefore reflected the actual stress during exercise more accurately.

The highest level of damage observed was mild to moderate, as classified by the extent and recovery time of strength loss. Mild muscle damage involves up to 20% strength reduction within the first 24 h, with recovery in 48 h, while moderate damage is a 20–50% reduction, recovering within 7 days^36^. The NP group showed approximately 20% peak torque reduction 24 h post-exercise, with near full recovery by 72 h, indicating mild to moderate damage. The LP group had a reduction of about 15%, fully recovering by 72 h, classifying it as mild. In contrast, the MP and HP groups showed almost no strength impairment the day after the intervention and beyond, indicating no EIMD.

#### Secondary damage markers

The VAS results align with the strength decrements, indicating that the NP group experienced the highest levels of muscle soreness, particularly 24 to 48 h after the intervention. Pain perception in the MP and HP groups remained consistently lower throughout the recovery period, while the LP group reported soreness levels comparable to NP, mirroring their similar strength reductions. Based on Boonstra et al.^47^, muscle soreness in both groups was also classified as mild, with peak VAS scores of 2.9 ± 2.5 for NP and 1.9 ± 1.9 for LP. These findings match previous research showing mild VAS ratings after BFR, comparable to but not higher than in free flow training^3,10,43,48^. If BFR training is performed with an AOP above 75%, it even seems to produce less DOMS than conventional LL training.

Furthermore, none of the secondary markers showed group differences over time. Our findings for CK and MB are consistent with studies showing no increase in these markers after BFR training^8,49,50^. While some studies reported elevated concentrations, they did not exceed those in control conditions^7,12,51^.The highest CK concentration in our study was 1717 U/L, well below the extreme cases exceeding 15,000 U/L reported by Sieljack et al.^12^. Similarly, we did not see prolonged edema following our intervention, consistent with previous findings^4,8,12^. Still, some studies have documented swelling following BFR training^6^. This indicates that swelling and biomarkers like CK and MB may not be sensitive enough to detect differences between BFR and non-BFR protocols when EIMD is mild to moderate. However, the significant PT reduction and VAS results emphasize the presence of EIMD, underscoring their importance in assessing EIMD.

### Acute exercise responses

To gain insight into the contribution of metabolic stress and mechanical work to muscle damage, we assessed SmO_2_, lactate, and the number of repetitions as acute exercise responses. These variables were included as indicators of stress, with the aim of explaining potential group-specific differences in EIMD responses. Given that both exercise-induced stressors are associated with muscle damage and inherently interrelated during exercise, these measures allowed us to estimate the shift and contribution of metabolic stress and mechanical work to EIMD under different AOPs.

#### Mechanical work

As expected, participants in the NP and LP groups completed more repetitions than the participants in the MP and HP groups and thereby accumulated higher mechanical work. This reduction in total workload in the latter groups aligns with previous data, as increasing AOP further restricts blood flow, reducing intramuscular oxygen delivery and venous clearance of metabolites^2^. The higher level of occlusion impaired recovery between sets by maintaining ischemic conditions during rest periods, delaying clearance of fatigue-related metabolites. As a result, metabolic by-products such as lactate and inorganic phosphate accumulated faster, contributing to an earlier onset of fatigue and a lower number of completed repetitions in the MP and HP groups. Consequently, these groups reached muscle failure almost twice as fast as the LP and NP groups.

#### SmO_2_

The inadequate oxygen supply with high AOP is supported by the SmO_2_ measurements that showed that muscle oxygen saturation was considerably lower in the HP group compared to the NP group. This is consistent with previous research indicating that AOPs above 75% results in lower SmO_2_ compared to LL training alone^52^. The continuous decline across sets and the lack of resaturation of SmO_2_ in the inter-set recovery periods also indicate that the blood flow restriction in HP prevented sufficient oxygenation even at rest. The body is thereby forced to increase its energy supply via anaerobic metabolism, which is supported by our spirometric data (SI Fig. 2), as the increase in RER was greater in the HP group compared to the NP group.

#### Perceived exertion

The higher proportion of anaerobic metabolism was also accompanied by higher BORG scores of the participants in the MP and HP groups compared to the NP group. As the participants were not informed about the AOP applied, the higher perceived exertion cannot be explained by anticipation or anxiety, which are known to influence the perception of exertion^53^. Rather, higher ratings are most likely due to a combination of the early transition to anaerobic metabolism, which intensifies the effort required for muscle recruitment and thus increases perceived exertion^54^ and the perception of intense pressure by skin nerves. The same finding was made in another study, which confirmed that BORG ratings tend to rise with increasing AOPs^55^. Interestingly, RPE values remained submaximal even with task failure. A possible reason for this could be the fact that cardiovascular load is reduced under BFR^56^. In addition, the isolated nature of single-leg knee extension may limit the applicability of RPE scales initially developed for dynamic whole-body exercise^57^. This discrepancy between high local fatigue and low systemic perception likely contributed to the observed submaximal RPE values.

#### Metabolic response

The SmO_2_ data indicated that the HP group finished the exercise with markedly lower muscle oxygen saturation, reflecting a greater local mismatch between oxygen supply and demand, likely due to the high degree of vascular occlusion. Despite this, post-exercise blood lactate concentrations did not increase with higher AOP. In fact, the LP group showed significantly higher average lactate concentrations than NP, suggesting greater systemic metabolic stress when occlusion pressure was low and exercise duration was longer. However, our findings show that lactate does not seem to explain differences in muscle damage. Instead, they indicate that raising the AOP does not increase the peak of metabolic stress, but rather shortens the duration of exposure to accumulating metabolites due to earlier onset of muscle failure.

While some studies report higher lactate concentrations at AOPs above 75% compared to low pressure or NP^52^, these studies used matched training volumes, which is a comparison between submaximal and maximal internal load^25^. When taken to concentric failure, lactate concentrations tend to equalize across conditions^38,48,58^. However, these measurements were all taken from systemic blood, rather than directly from the muscle tissue, where lactate could accumulate during restricted blood flow before entering circulation. Yet, Franz et al.^42^ compared intramuscular lactate concentrations between LL resistance training with 50% AOP and without BFR to muscle failure. They have also been unable to detect differences during exercise and up to one hour post-exercise between the two conditions. Given that intramuscular and blood lactate concentrations at LP are similar to non-BFR training, coupled with a shorter workout duration, it could be argued that the overall metabolic load is potentially even lower with BFR. Whether this also applies for higher AOPs remains to be investigated, as intracellular lactate concentrations may reach their peak more quickly due to impaired clearance, resulting in rapid rather than gradual accumulation.

### Possible causes of muscle damage

Usually, during high load eccentric exercise, high tensile forces disrupt structural proteins, especially at weakened z-lines, and impose excessive strain on connective tissue at the myotendinous junction and muscle fibers^30,32,59^. Even at low loads, damage can occur due to hypoxia and ATP depletion, which contribute to muscle membrane damage during low-force contractions^20^. In our study, however, high forces were not applied, and the magnitude of metabolic stress appeared comparable across groups. Yet, moderate to mild damage occurred under low load conditions with low AOP and no BFR. This is explained by the fact that although low-intensity contractions typically cause minimal damage^60^, an increased number of lengthening contractions can elevate EIMD^61,62^. It is likely that this accounts for the similar strength impairments observed in the NP and LP groups, which were exposed to comparable mechanical work. Conversely, the MP and HP groups, which faced lower mechanical work, exhibited less muscle damage.

As mentioned before, it is important to recognize that metabolic stress and mechanical work cannot be entirely separated during exercise. However, we did not observe these two mediators existing on a continuum regarding their relative weighting during exercise to failure, as previously suggested. Shifts to AOPs above 75% lead to a reduction in mechanical work, but not to an increase in metabolic stress, at least systemically. In addition to mechanical work, total time under occlusion should also be considered, as research suggests that BFR-induced stress responses are time-dependent with longer occlusion periods potentially contributing to greater cellular damage^63^. In our study, the NP and LP groups exercised twice as long as MP and HP. Although post-hoc analysis revealed that average lactate concentration post-exercise was higher in the LP group compared to the NP group, the MP and HP groups reached similar lactate concentrations despite exercising for a significantly shorter duration. This suggests that while the peak metabolic load was comparable, the overall metabolic strain in MP and HP was likely lower due to the reduced duration of exposure. However, as mentioned in the Metabolic Response chapter, the kinetics at different pressures still need to be examined intramuscularly during exercise, as higher pressures may reach their peak faster due to impaired clearance, which could lead to rapid rather than gradual accumulation. The short occlusion time in MP and HP may therefore have limited not only the mechanical load but also the build-up of metabolic stress beyond physiological thresholds, possibly also contributing to the low EIMD values observed.

This suggests that while metabolic stress may contribute to muscle damage, its impact could be less direct than anticipated. In practical exercises where training is performed to or near exhaustion, both the peak and the duration of the metabolic stress that can affect the tissue appear to be limited by the onset of fatigue. Consequently, the total amount of mechanical stress applied prior to fatigue is probably more critical for the development of EIMD, either through high tensile forces beyond the fiber capacity, as seen in eccentric training, or from sufficient accumulative low-intensity contractions combined with training near muscle failure.

## Limitations

Several limitations to our study should be acknowledged. Firstly, participants’ physical activity and dietary intake outside of the study sessions were not standardized or closely monitored. While participants were instructed to maintain their usual habits and refrain from engaging in additional or unaccustomed exercise, we cannot entirely rule out the potential influence of unreported activity or nutritional differences on the extent of muscle damage or recovery. This may have introduced variability in the outcome measures and should be considered when interpreting the results.

Another limitation is the variability in CK and MB concentrations. In the HP group, a few outliers exhibited elevated CK and MB concentrations at baseline, as reflected by the high standard deviations. However, since these concentrations did not change over the study period, it is unlikely that these participants were pre-damaged. Instead, this underlines the high variability in blood markers, indicating that this parameter can only be accurately assessed from an individual long-term perspective^64^.

We also acknowledge that blood flow was not measured directly during the experiment. While we did monitor local muscle oxygenation via near-infrared spectroscopy (NIRS) during the intervention, we recognize that this method does not provide a direct quantification of arterial inflow or venous outflow. Therefore, the actual degree of blood flow restriction remains uncertain and may have influenced the observed outcomes.

Furthermore, although the study was generally successful in differentiating the mechanical work between the groups, the gradation was not as distinct as intended. The MP and HP groups experienced almost identical mechanical work, suggesting that the difference between 75 and 100% AOP may have minimal effect and that there is no clear linear relationship between AOP and changes in arterial blood flow. Our initial assumption that the number of repetitions is reduced at higher AOPs was based on evidence that recovery between sets is increasingly impaired with increasing pressure, rather than greater deoxygenation during contractions. Reis et al.^65^ showed that deoxygenation plateaued between 60 and 80% AOP, likely because muscle contractions already limit perfusion through capillary compression. However, reoxygenation during rest was significantly reduced at higher pressures, indicating impaired recovery capacity. While this supported our expectation of earlier fatigue at higher pressures, our data did not confirm this, indicating that performance differences beyond a certain AOP may reach indeed a plateau or ceiling effect, with any further impact too marginal to be reflected in repetition count. In contrast, the LP group showed a large variance in mechanical work, suggesting that some participants may have stopped the intervention due to discomfort rather than fatigue. In general, the LP group showed a significant reduction in peak torque one hour after exercise, suggesting that neuromuscular fatigue was indeed present. However, given the variation in performance, it remains plausible that individual differences in tolerance contributed to premature termination in some participants, rather than representing a general trend within the group. The absence of a time-matched control group should also be acknowledged. Because exercise duration differed between conditions, we cannot fully isolate the effects of occlusion pressure from total training time. However, using time-matched protocols would have resulted in different internal loads across groups, which would have conflicted with our primary aim of comparing responses under equal internal load conditions achieved by training to or near failure.

Lastly, it is important to note that although participants were not given any information for their assigned percentage of AOP, effective blinding is not truly achievable when using BFR as participants can feel the pressure.

## Practical applications

In conclusion, our randomized controlled trial indicates that BFR does not increase muscle damage compared to conventional LL training, regardless of the applied AOP. In fact, participants in the MP and HP groups showed virtually no EIMD, as evidenced by a negligible reduction in strength and essentially no DOMS. Moreover, the highest degree of EIMD observed in our study was mild in the LP group, emphasizing the safety of BFR training.

From a practical perspective, AOPs above 75% did not induce muscle damage, yet this was accompanied by a substantial reduction in total mechanical work. The potential implications of these acute responses for long-term adaptations remain to be investigated.

This concern is less relevant in LP BFR training, where mechanical work is comparable to conventional LL training. Therefore, training with 50% of AOP appears to be a safe and time-efficient method for young, healthy adults, offering a good balance between training effectiveness and a limited risk of mild muscle damage when applied appropriately.

## Electronic supplementary material

Below is the link to the electronic supplementary material.

Supplementary Material 1

## Data Availability

The data that support the findings of this study are not openly available due to reasons of sensitivity and are available from the corresponding author upon reasonable request.

## Abbreviations

AOP: Arterial occlusion pressure
BFR: Blood flow restriction
CK: Creatine kinase
D_m_: Maximal displacement
DOMS: Delayed-onset muscle soreness
EIMD: Exercise-induced muscle damage
LL: Low load
MB: Myoglobin
NIRS: Near-infrared spectroscopy
PT: Peak torque
RER: Respiratory exchange ratio
RPE: Rate of perceived exertion
ROS: Reactive oxygen species
SmO_2_: Oxygen saturation
SWE: Shear-wave elastography
T_c_: Contraction time
TMG: Tensiomyography
US: Ultrasound
VAS: Visual analog scale
