# Supplementary material for: Effects of different arterial occlusion pressures during blood flow restriction exercise on muscle damage: a single-blind randomized controlled trial
Source: Sci Rep. 2025 Jul 31;15:27985. doi: 10.1038/s41598-025-11654-y (PMC12313878; doi:10.1038/s41598-025-11654-y)
Supplement: Supplementary file 1 — Supplementary Material 1 [file 41598_2025_11654_MOESM1_ESM.docx]

# **Supplementary Material**


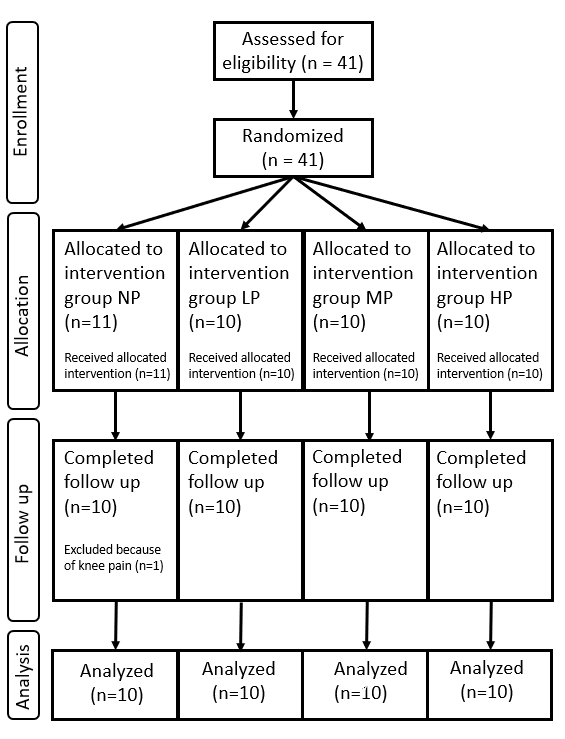


***SI Figure 1***.: Schematic flowchart of each study phase, including number of participants. NP = no pressure, LP = low pressure, MP = medium pressure, HP = high pressure.

**Supplementary Methods**

**Muscle stiffness**

To assess muscle stiffness, the position of the participants was identical to the one described for the muscle thickness measurement. The same Siemens ACUSON Redwood system (Siemens Healthineers) was used, equipped with a 10L4 linear array probe (50 mm wide). The additional markings (± 25 mm) indicated the measurement spot. As required in SWE techniques, the ultrasound probe was aligned in the longitudinal direction of the muscle fiber [3]. Accordingly, the probe was placed with minimal pressure along the direction of the muscle fibers within the 50 mm mark, matching the probe width. The region of interest measured 25 x 15 mm, with its upper edge aligned with the upper fascial sheath of the muscle. Within this region, elastography values ranging from 0.5 to 6.5 m/s were analyzed in two measurement circles, each 10 mm in diameter. These circles (lesions) were placed one below the other, starting at the fascial border. The same approach was used for the m. VL, with the upper lesion (L3) and the lower lesion (L4) placed in a similar manner. Each lesion was checked for quality by using the real-time feedback confidence map. This color-coded overlay shows the regions where the system has high or low confidence in the accuracy of the stiffness measurements, ensuring reliability before reporting or proceeding with diagnosis. Every measurement was performed three times and the mean used for further analysis. For the RF, L1 and L2 were averaged to calculate the stiffness value (SWE RF). For the VL, L3 and L4 were averaged (SWE VL). The average of all four lesions provided the total stiffness value in m/s (SWE). Images were stored at each measurement to serve as benchmarks for subsequent assessments, enhancing between-day repeatability. Anatomical landmarks were used as reference markers for setting regions of interest. The experimenter conducting the measurements was blinded to group allocation.

**Thermography**

After a 15-minute acclimatization in a laboratory room at a standardized room temperature of 21-23 degrees, thermography of the front of the thigh was conducted from a distance of one meter using a thermal camera (FLIR E86-EST thermal camera, FLIR Systems inc., USA). All images were analyzed in the FLIR ResearchIR Standard 4 software (FLIR Systems, USA) and the average temperature of the thigh was determined.

**Muscle contractility**

The previously mentioned markings and additional lines also served as references for the sensor and electrode placement during the Tensiomyography (TMG) measurement, ensuring consistent positioning. The sensor was placed perpendicular to the tangential plane on the marked area over the muscle belly. The stimulation protocol involved delivering a new stimulus every 30 seconds in 10mA increments, starting at 70mA, until either the maximum detectable amplitude of radial displacement or 110mA was reached. Participants were instructed to relax as much as possible during the test. The trial with the largest maximum radial displacement (D_m_) was used for further evaluation.

**Respiratory gas exchange**

During training, the respiratory exchange ratio (RER) was analyzed using Cosmed Quark CPET spirometry (CosMed GmbH & Co. KG). After calibration, spirometry was applied approximately 10 minutes before the intervention and participants were instructed to remain as quiet as possible to avoid influencing the measurements. RER was calculated as the mean over the last minute prior to the intervention and the last minute immediately post-intervention. RER was then analyzed as the change (Δ) from baseline (pre-intervention) to the end of each individual intervention session. The spirometric data, which focused on the ratio of carbon dioxide produced to oxygen consumed (RER), provided insights into the metabolic responses during the exercise.

**Supplementary statistical analysis**

We used repeated measures ANCOVAs with baseline as a covariate to analyze the triple interaction effect of group x time x baseline on secondary damage markers as an exploratory approach. However, we refrained from using this approach for the primary outcomes, as it is a relatively uncommon method. Given the robust validation and widespread use of change scores in the literature, we opted to align our primary outcomes with this established method. This ensures that our findings can be accurately compared within the context of existing research, thereby enhancing their relevance and impact.

We conducted all assumption tests for the statistical methods employed and applied the necessary corrections where required. Specifically, the Welch test was utilized when Levene's test indicated inhomogeneous variance for one-way ANOVA. For repeated measures ANOVA, the Greenhouse-Geisser correction was applied if Mauchly's test indicated a violation of sphericity.

**Supplementary Results**

***SI Table 1***.: Descriptive data of participants.

| **Parameter** | **NP**  **(n=10)** | **LP**  **(n=10)** | **MP**  **(n=10)** | **HP**  **(n=10)** | **Total**  **(n=40)** |
| --- | --- | --- | --- | --- | --- |
| **RER** | 0.95 ± 0.03 | 0.89 ± 0.02 | 0.97 ± 0.02 | 0.89 ± 0.04 | 0.92 ± 0.09 |
| **Skintemp. (°C)** | 29.3 ± 2.27 | 29.6 ± 1.27 | 28.9 ± 1.03 | 29.8 ±1.28 | 29.4 ± 1.51 |
| **SWE RF (m/s)** | 1.8 ± 0.20 | 1.6 ± 0.23 | 1.8 ± 0.32 | 1.8 ± 0.25 | 1.8 ± 0.25 |
| **SWE VL (m/s)** | 1.7 ± 0.21 | 1.7 ± 1.54 | 1.5 ± 0.18 | 1.6 ± 1.50 | 1.6 ± 0.18 |
| **SWE (m/s)** | 1.7 ± 0.21 | 1.7 ± 0.17 | 1.6 ± 0.25 | 1.7 ± 1.40 | 1.7 ± 0.19 |
| T_c_**RF (ms)** | 29.2 ± 4.20 | 26 ± 3.77 | 32.7 ± 5.55 | 29.4 ± 5.17 | 29.3 ± 5.15 |
| D_m_**RF (mm)** | 7.1 ± 1.46 | 6.9 ± 1.46 | 8.9 ± 2.30 | 6.9 ± 5.69 | 7.5 ± 3.88 |
| T_c_**VL (ms)** | 22.5 ± 3.81 | 23.4 ± 4.06 | 25.9 ± 6.22 | 23.7 ± 5.80 | 23.9 ± 4.68 |
| D_m_**VL (mm)** | 7.4 ± 2.61 | 5.4 ± 3.25 | 6.5 ± 2.71 | 6.2 ± 1.89 | 6.4 ± 2.42 |

The data presented are in means ± SD; NP = no pressure; LP = low pressure; MP = medium pressure; HP = high pressure; RER = respiratory exchange ratio; RF = rectus femoris; VL = vastus lateralis; TH = muscle thickness; SWE = shear-wave elastography; T_c_ = contraction time; D_m_ = maximal displacement

**Respiratory gas exchange**

The Welch's test showed an effect of applied AOP on RER elevation (Δ to baseline) from the end of training to 3 min after the intervention [F(3, 18.51) = 4.16, p = 0.021, η² = .280]. Multiple comparisons found a difference between NP and HP (MD = -26.97, SE = 7.82, p = .0176, 95% CI [-50.03, -3.89]).


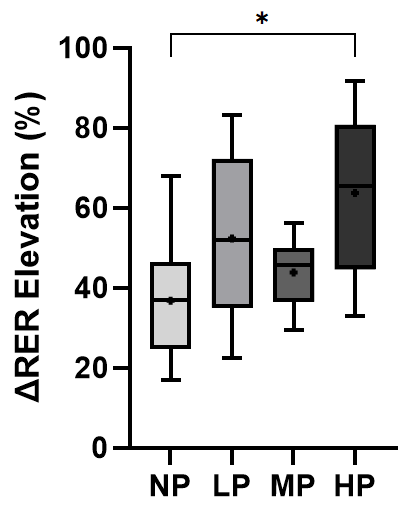
Thus, these results show that HP participants **(**M = 63.72 **±** 19.67 SD) provided more of the energy requirements via anaerobic metabolism compared to NP participants **(**M = 36.75 **±** 15.00 SD) (Figure 2D).

***SI Figure 2***.: Group differences during intervention. Changes of respiratory exchange ratio from baseline to the end of intervention. Box and whisker plots show the median (line within the box), the interquartile range (box, spanning Q1 to Q3), and the minimum and maximum values (whiskers). The dot in the middle represents the mean. RER = respiratory exchange ratio, NP = no pressure, LP = low pressure, MP = medium pressure, HP = high pressure. * = significant differences of p < .05.

**Skin temperature**

Results revealed no main effect of time [F(2.02, 64.68) = 1.44, p = .244, partial η² = .044], as well as a main effect of group [F (3, 32) = 2.32, p = .095, partial η² = .183] for skin temperature. Neither the interaction effect between time and group [F(6.06, 64.68) = 0.96, p = .459, partial η² = .085] and three-way interaction effect of time, group, and baseline skin temperature: [F(8.08, 64.68) = 0.87, p = .555, partial η² = .100].

These results indicate that there were no changes in skin temperature over time between 1 minute and 10 minutes after the intervention and no differences between groups. Despite no time effects being observed when baseline values were used as a covariate, it is again important to note that skin temperature showed a substantial and consistent increase across all groups following the intervention, reflecting temperature stability immediately after the intervention. The mean pre-intervention skin temperature was 29.43° ± 0.76. It increased to 31.42° ± 0.77 at 1-minute post-intervention and remained elevated at 31.34° ± 0.75 at 3 minutes, 31.73° ± 0.77 at 7 minutes, and 31.89° ± 0.64 at 10 minutes.

***SI Table 2***.: Results of repeated measures ANOVAs from secondary damage markers.

| Parameter | Group | | | Time | | | Group x time | | | Group x time x baseline | | |
| --- | --- | --- | --- | --- | --- | --- | --- | --- | --- | --- | --- | --- |
|  | F | p | η² | F | p | η² | F | p | η² | F | p | η² |
| Thermography | 0.76 | .524 | .071 | 6.13 | **< .001** | .170 | 1.51 | .157 | .131 | 2.25 | **.015** | .231 |
| Stiffness total | 1.45 | .233 | .048 | 2.67 | .066 | .216 | 1.31 | .241 | .120 | 1.34 | .214 | .155 |
| Stiffness RF | 1.17 | .340 | .123 | 1.01 | .392 | .039 | .88 | .549 | .095 | 1.09 | .377 | .149 |
| Stiffness VL | 2.77 | .107 | .090 | .601 | .616 | .021 | .42 | .888 | .043 | 0.39 | .944 | .052 |
| T_c_ RF | 9.77 | **< .001** | .610 | 2.67 | .066 | .216 | 1.37 | .215 | .128 | 1.07 | .130 | .133 |
| T_c_ VL | 2.55 | .**026** | .545 | 1.13 | .353 | .112 | 1.44 | .203 | .138 | 1.53 | .153 | .185 |
| D_m_ RF | 9.79 | **< .001** | .502 | 0.09 | .953 | .003 | 2.94 | **.009** | .234 | 1.77 | .072 | .195 |
| D_m_ VL | 0.40 | .755 | .044 | 0.29 | .830 | .011 | 0.61 | .777 | .065 | 0.52 | .877 | .074 |

CK = creatine kinase; RF = rectus femoris; VL = vastus lateralis; T_c_ = contraction time; D_m_= maximal displacement

**Supplementary Discussion**

Further insights were given by TMG, which is used to assess muscle contractility and damage [5]. While no prior study has used TMG to evaluate EIMD after BFR training, one study reported no differences in contractility adaptations between BFR and non-BFR after six weeks [7]. In our study, we also observed no acute differences in D_m_ or T_c_ between groups over the 72-hour period.

We additionally investigated muscle damage using SWE. One study compared muscle stiffness adaptations between BFR and non-BFR training, as well as high-load training over six weeks, and found no differences in stiffness between conditions [6]. Similarly, our study found no differences in acute muscle stiffness changes over time across different AOPs in LL training.

Finally, we used thermal imaging to assess muscle responses across protocols. Although this method has been shown to identify both local [2] and systemic muscle damage [8], caution is advised when interpreting skin temperature as a marker of muscle damage [4]. Our initial results suggested a triple interaction effect, but post-hoc analysis revealed no differences between groups. This is consistent with a study that found no thermal changes between BFR and non-BFR training after 24 hours [1].

**Supplementary Limitation**

Due to the extensive time required per participant, data collection took place on a weekly basis, resulting to variations in environmental conditions across different seasons which made standardization for thermal imaging challenging.

**
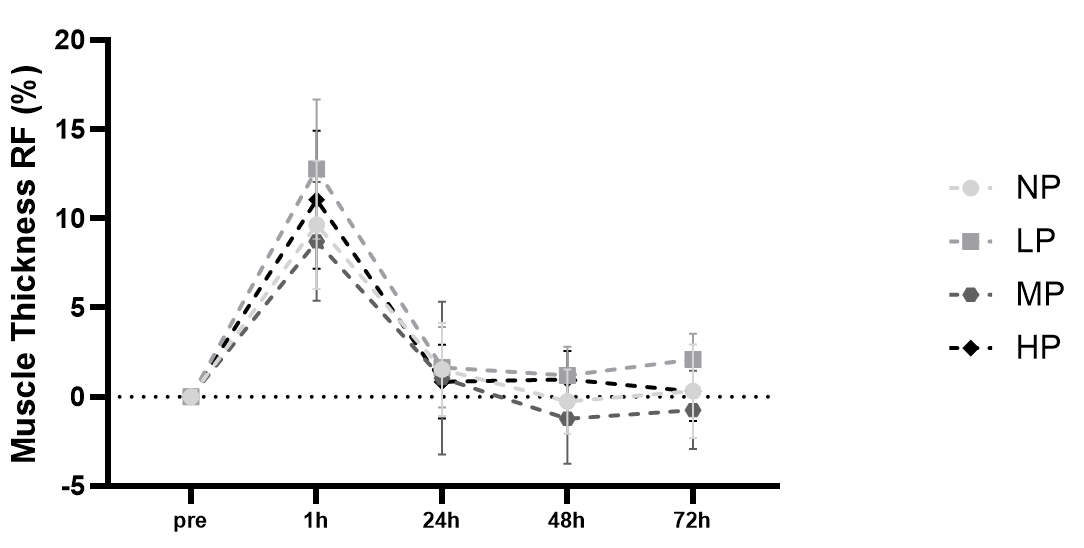
*SI Figure 3***.: Comparison of exercise-induced muscle thickness changes of the rectus femoris from baseline to 1h, 24h, 48h and 72h post-exercise (mean with 95% CI). NP = no pressure, LP = low pressure, MP = medium pressure, HP = high pressure

**
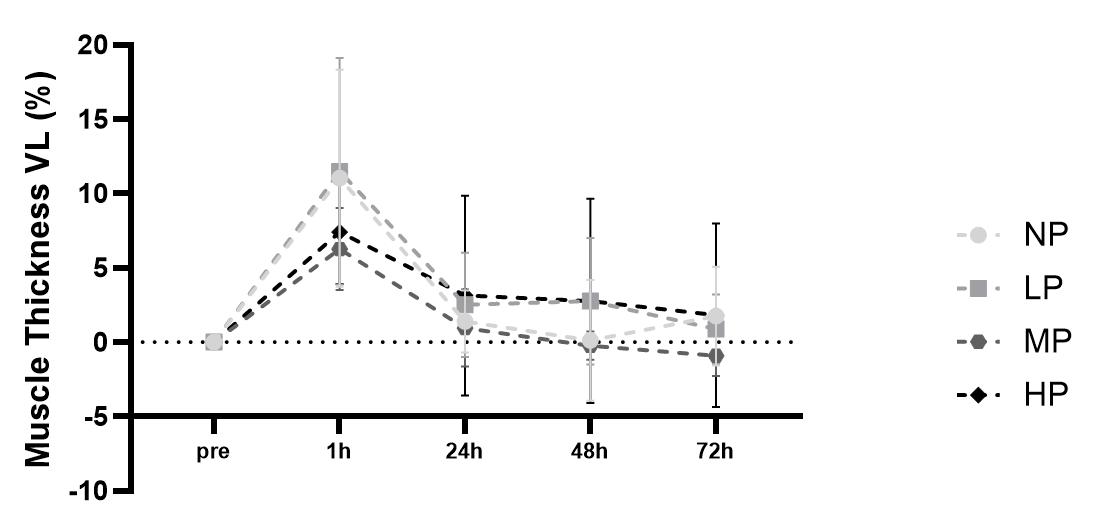
**

***SI Figure 4***.: Comparison of exercise-induced muscle thickness changes of the vastus lateralis from baseline to 1h, 24h, 48h and 72h post-exercise (mean with 95% CI). NP = no pressure, LP = low pressure, MP = medium pressure, HP = high pressure

**
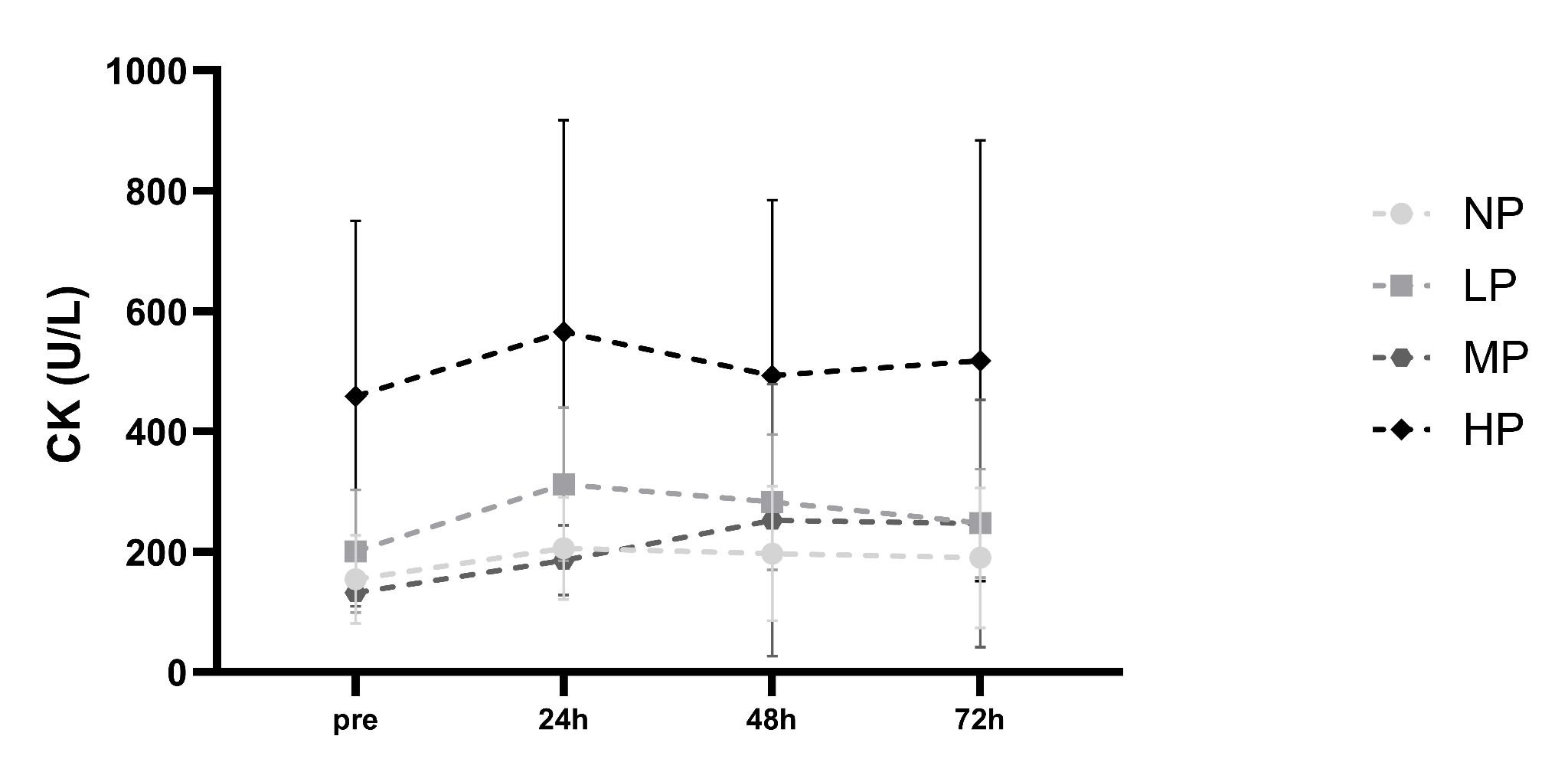
**

***SI Figure 5***.: Comparison of creatine kinase concentrations at pre, 24h, 48h and 72h post-exercise (mean with 95% CI). NP = no pressure, LP = low pressure, MP = medium pressure, HP = high pressure

**
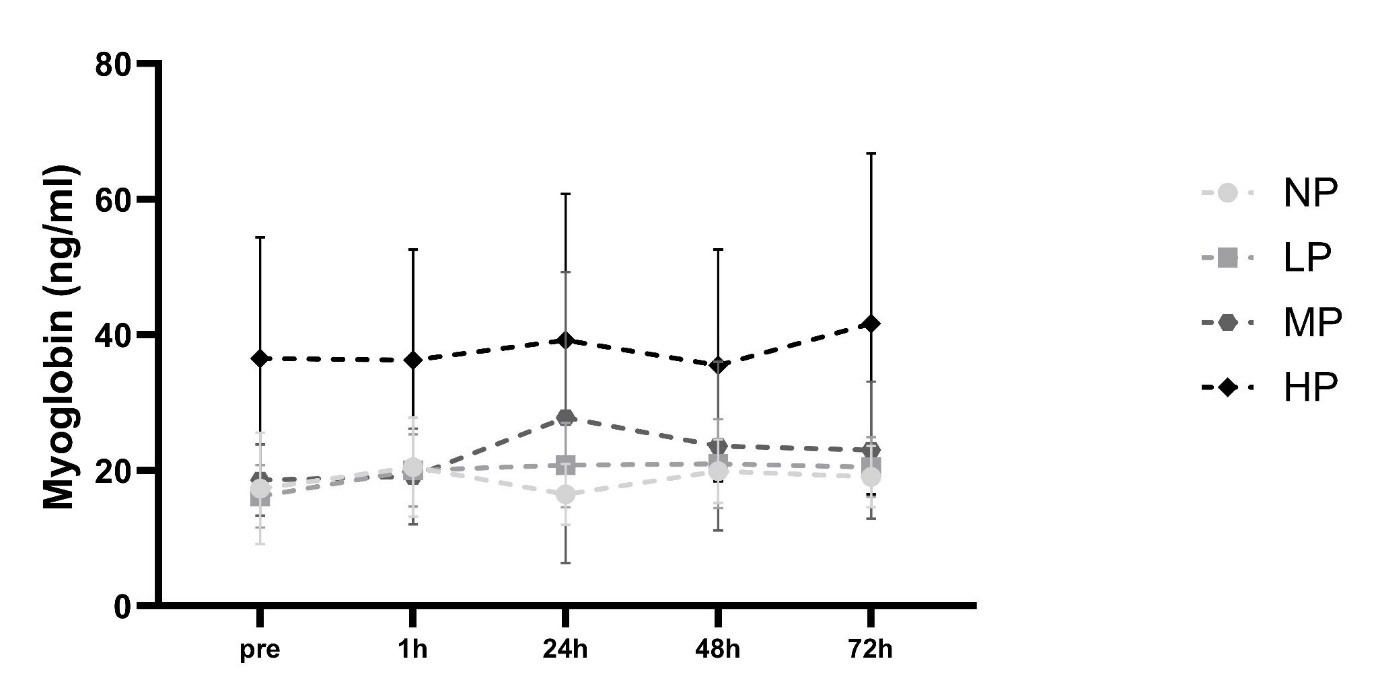
**

***SI Figure 6***.: Comparison of myoglobin concentrations at pre, 1h, 24h, 48h and 72h post-exercise (mean with 95% CI). NP = no pressure, LP = low pressure, MP = medium pressure, HP = high pressure

**
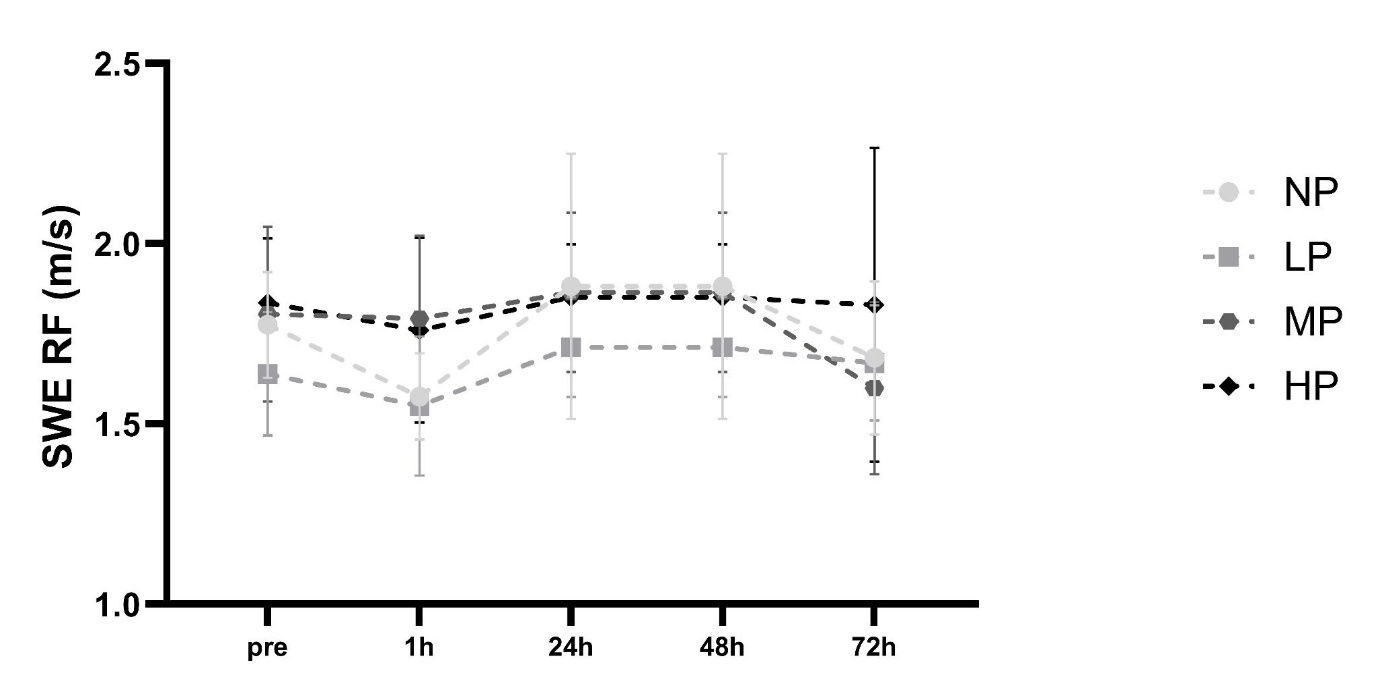
**

***SI Figure 7***.: Comparison of rectus femoris muscle stiffness at pre, 1h, 24h, 48h and 72h post-exercise (mean with 95% CI). NP = no pressure, LP = low pressure, MP = medium pressure, HP = high pressure

**
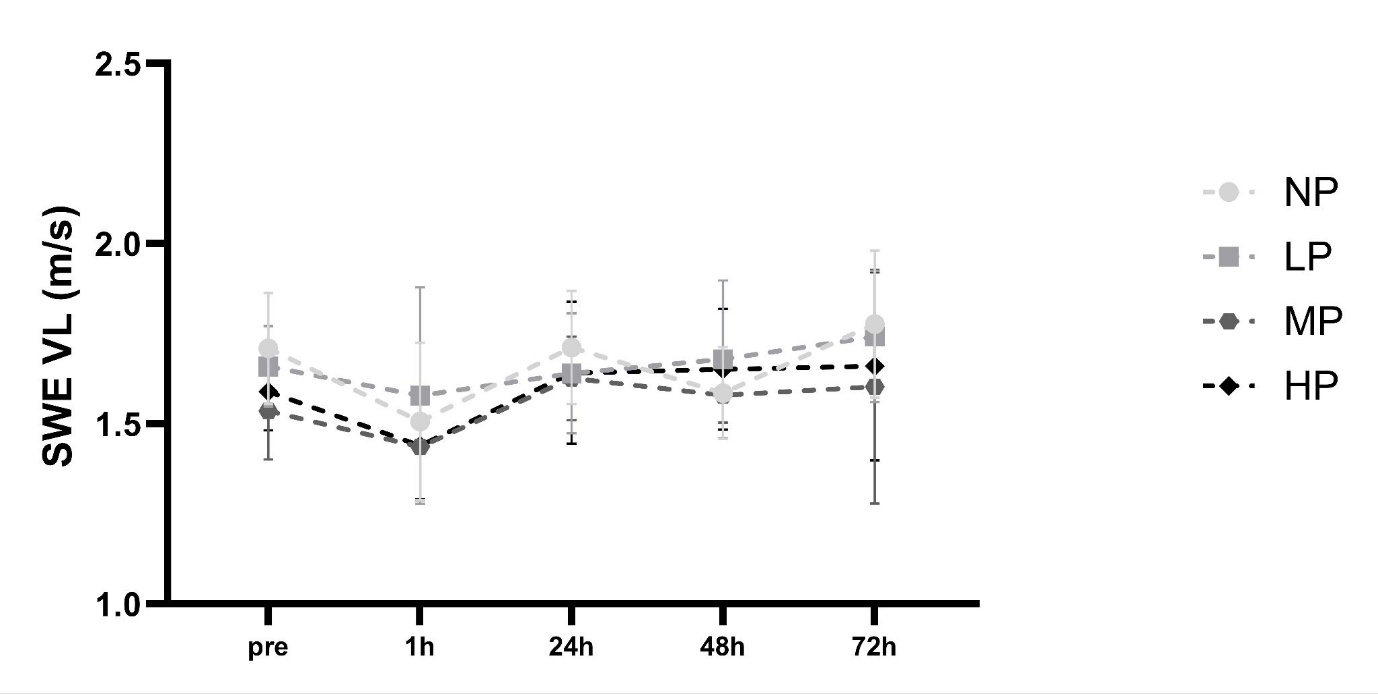
**

***SI Figure 8***.: Comparison of vastus lateralis muscle stiffness at pre, 1h, 24h, 48h and 72h post-exercise (mean with 95% CI). NP = no pressure, LP = low pressure, MP = medium pressure, HP = high pressure

**
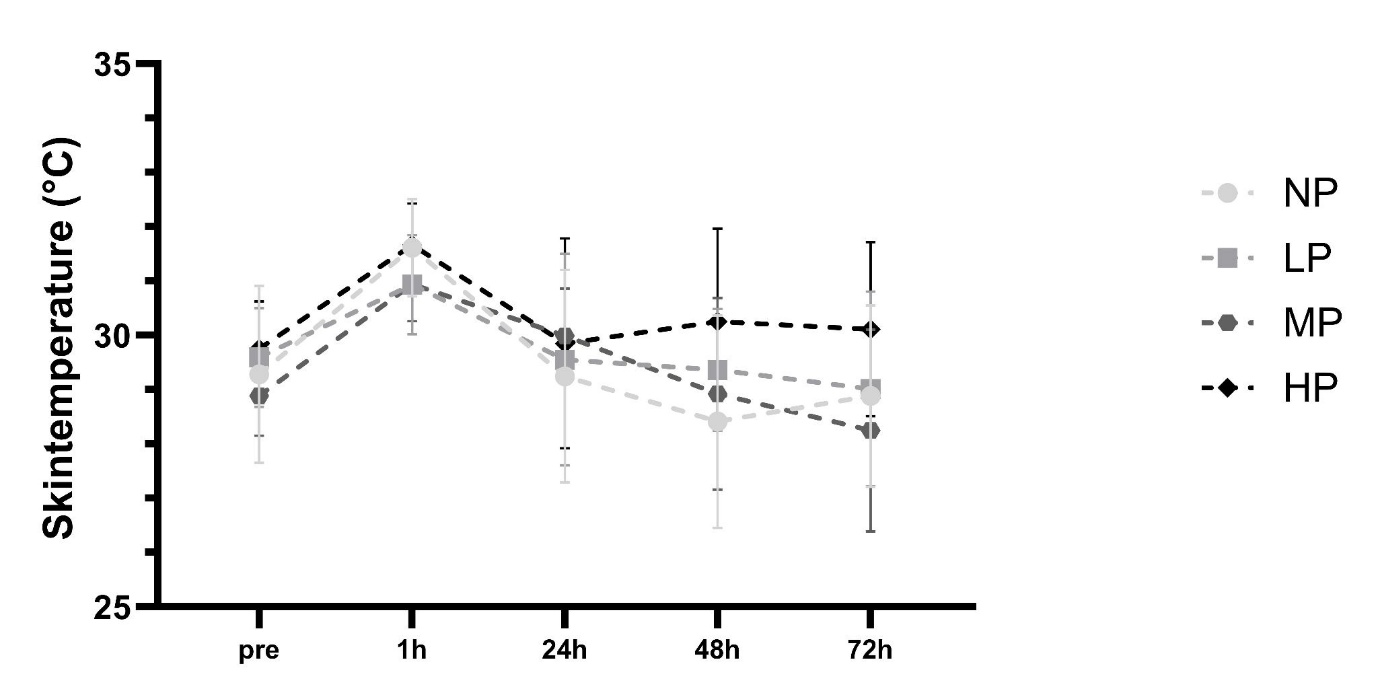

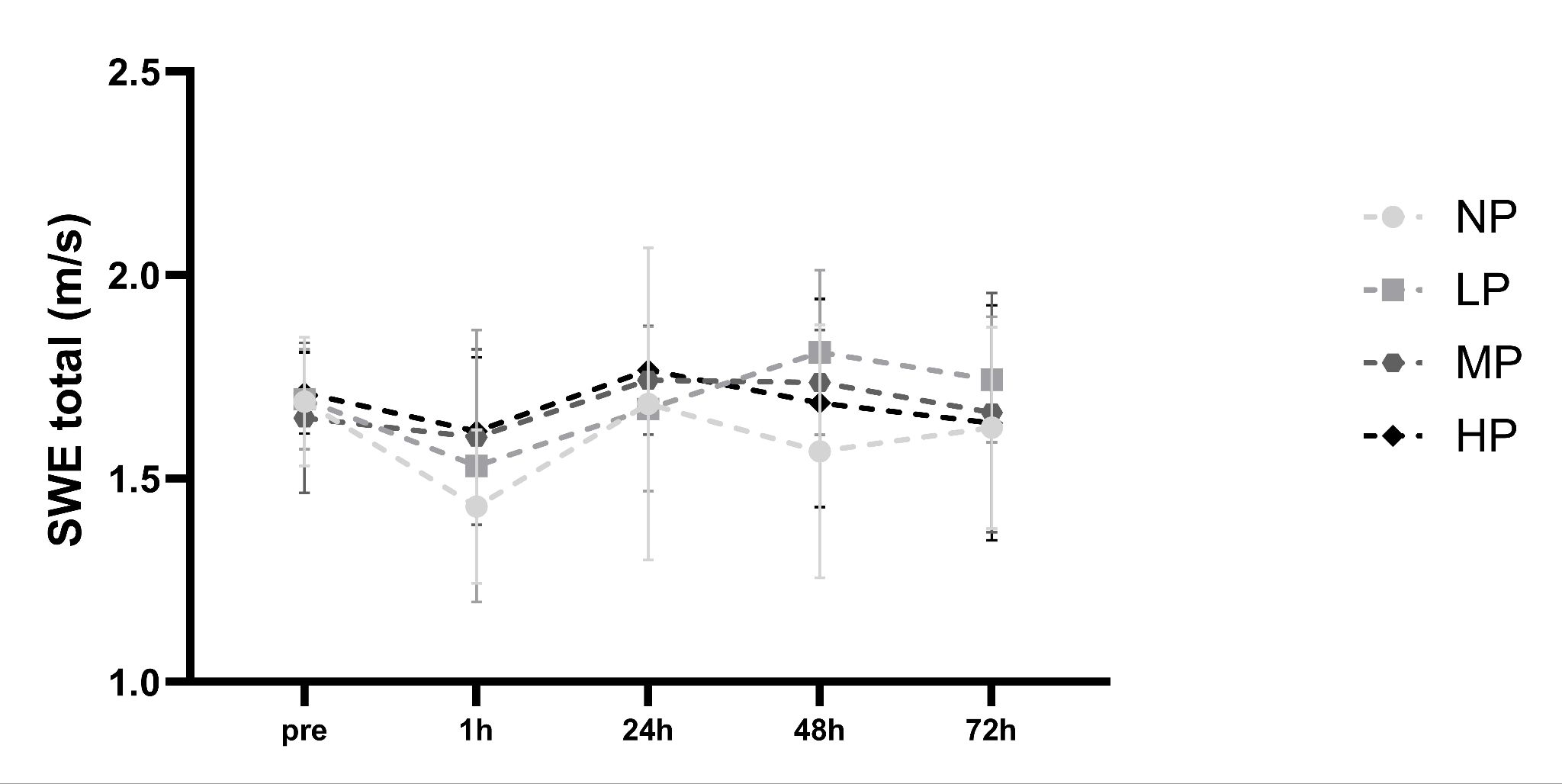
**

***SI Figure 9***.: Comparison of total muscle stiffness at pre, 1h, 24h, 48h and 72h post-exercise (mean with 95% CI). NP = no pressure, LP = low pressure, MP = medium pressure, HP = high pressure

***SI Figure 10***.: Comparison of skintemperatur at pre, 1h, 24h, 48h and 72h post-exercise (mean with 95% CI). NP = no pressure, LP = low pressure, MP = medium pressure, HP = high pressure

**
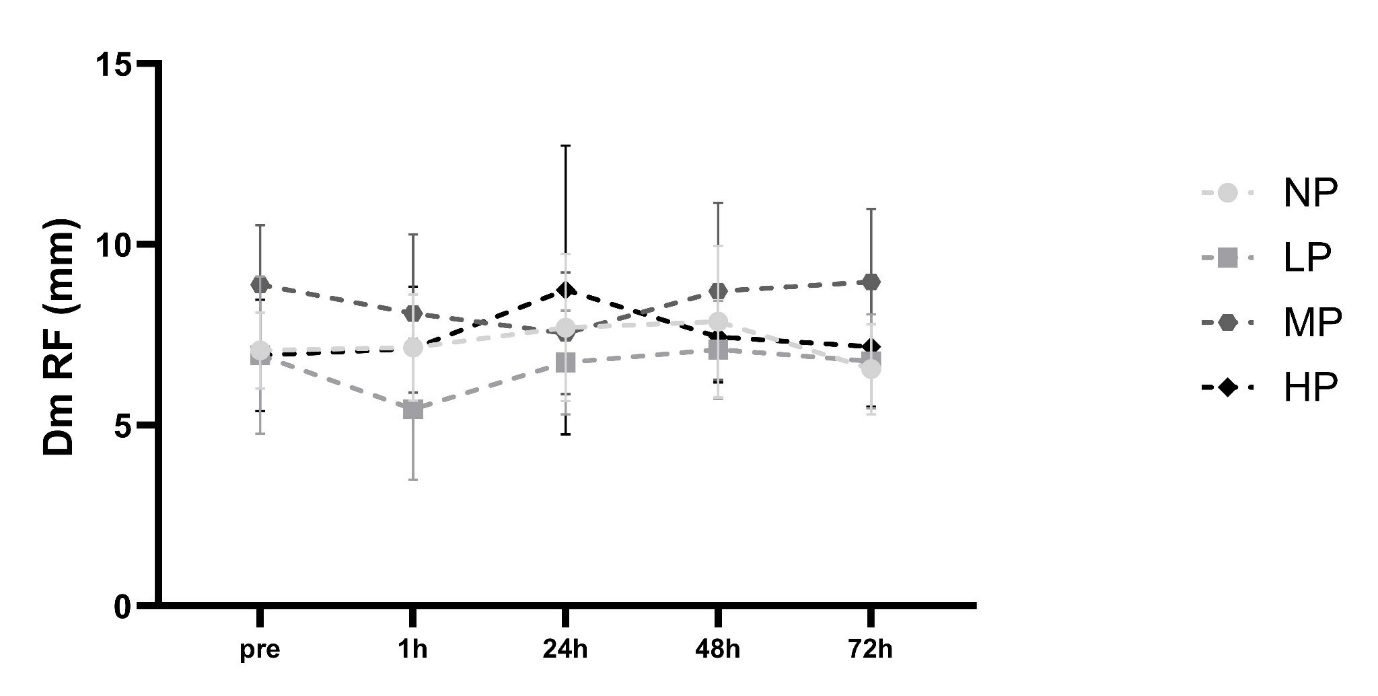
**

***SI Figure 11***.: Comparison of rectus femoris maximal displacement at pre, 1h, 24h, 48h and 72h post-exercise (mean with 95% CI). NP = no pressure, LP = low pressure, MP = medium pressure, HP = high pressure

**
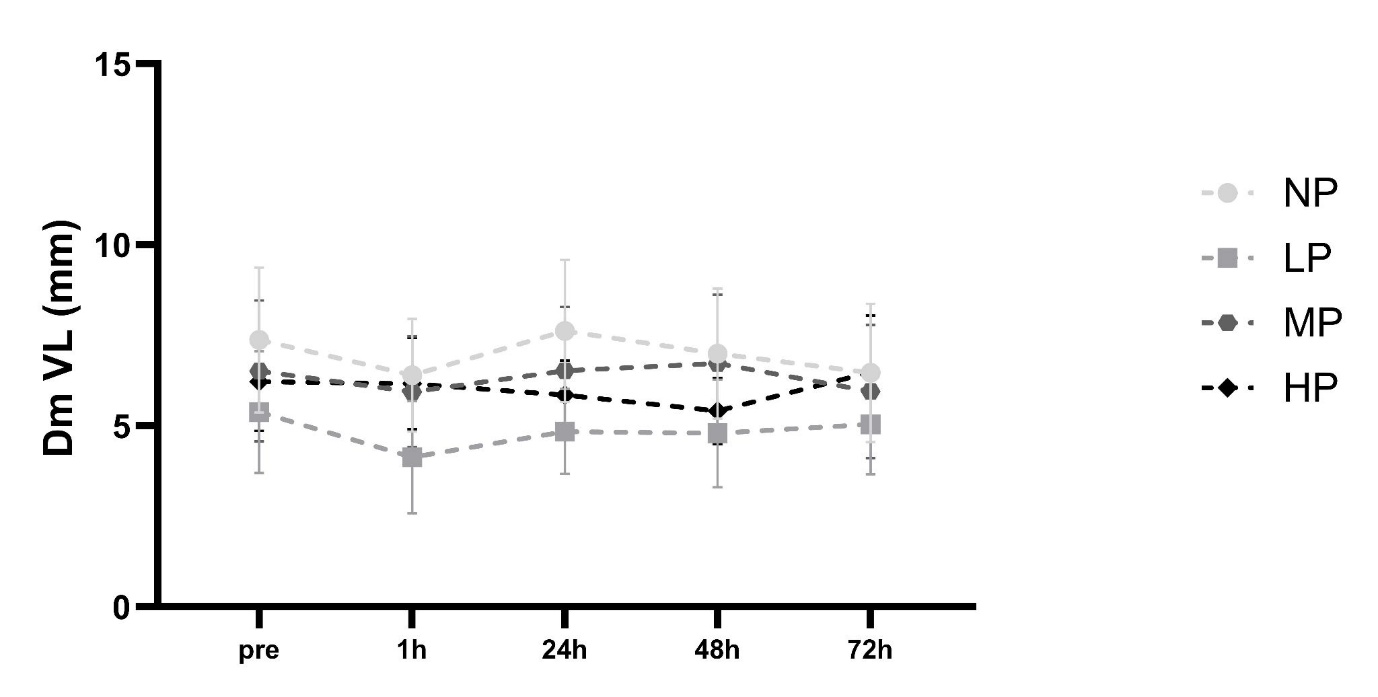
**

***SI Figure 12***.: Comparison of vastus lateralis maximal displacement at pre, 1h, 24h, 48h and 72h post-exercise (mean with 95% CI). NP = no pressure, LP = low pressure, MP = medium pressure, HP = high pressure

**
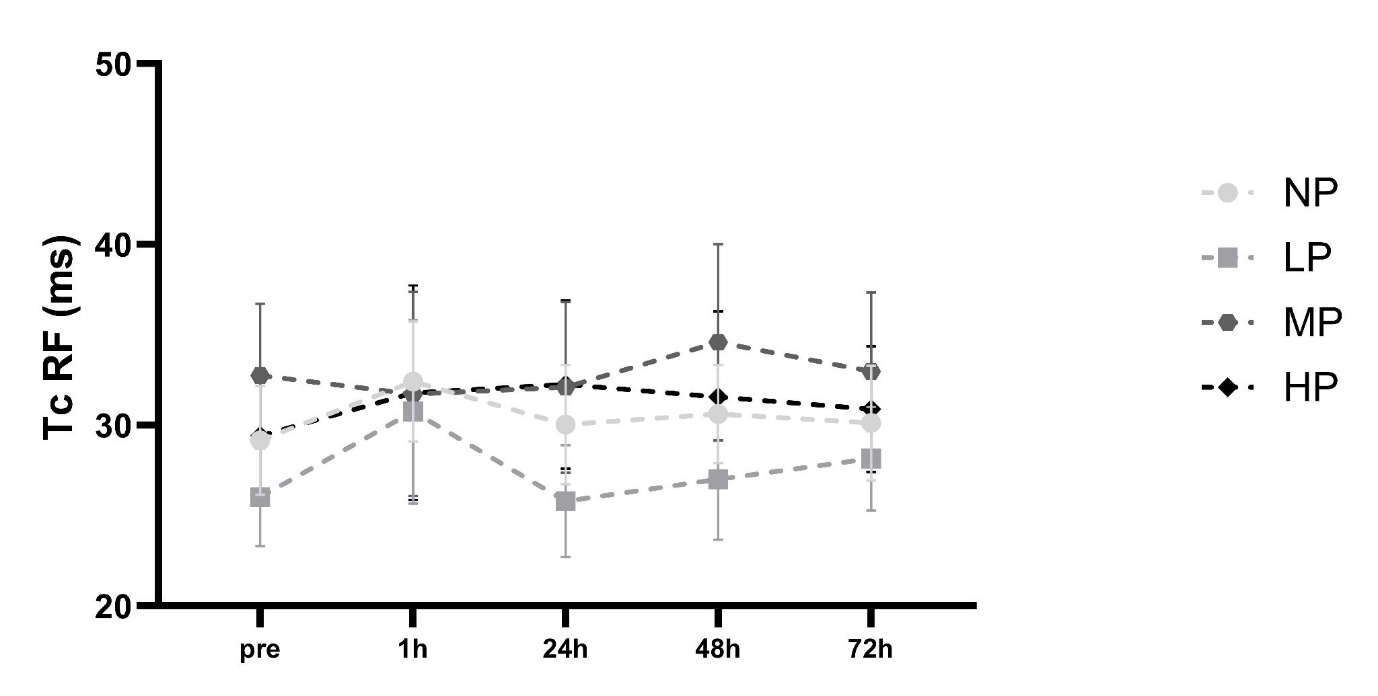
**

***SI Figure 13***.: Comparison of rectus femoris contraction time at pre, 1h, 24h, 48h and 72h post-exercise (mean with 95% CI). NP = no pressure, LP = low pressure, MP = medium pressure, HP = high pressure

**
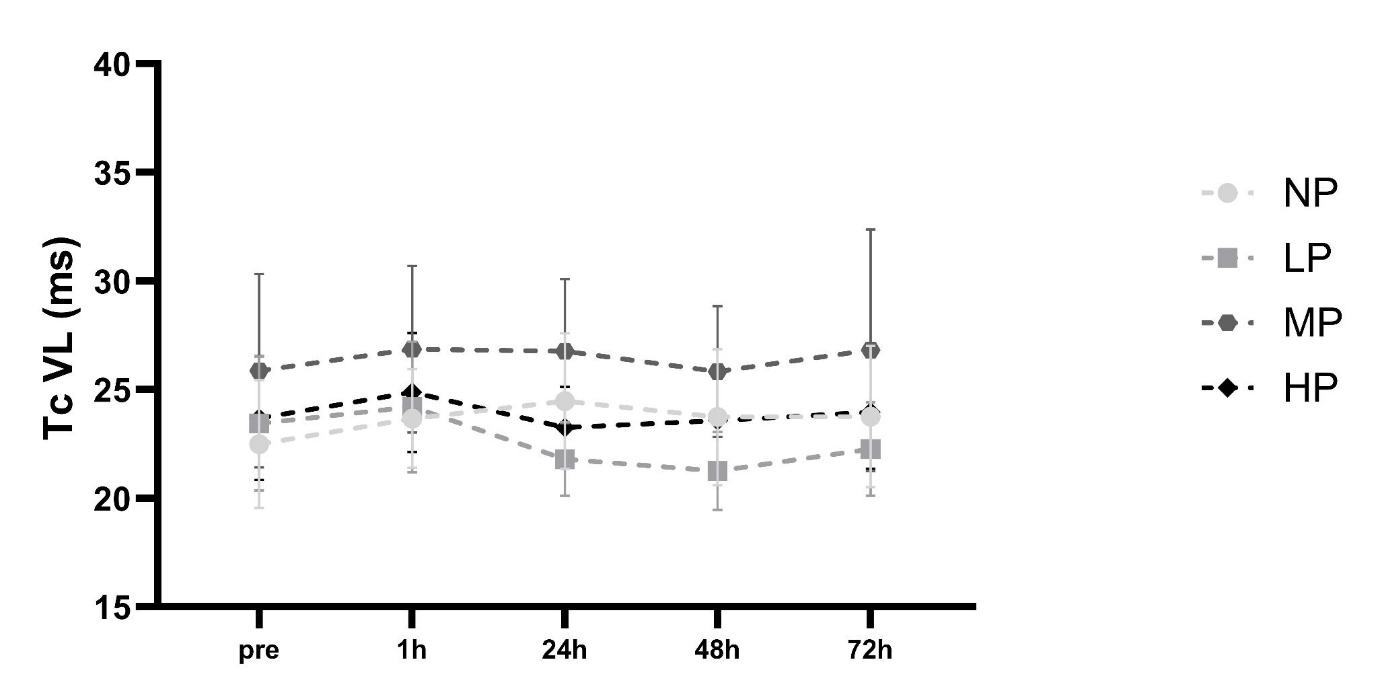
**

***SI Figure 14***.: Comparison of vastus lateralis contraction time at pre, 1h, 24h, 48h and 72h post-exercise (mean with 95% CI). NP = no pressure, LP = low pressure, MP = medium pressure, HP = high pressure

References

[1] ALMEIDA BARROS, N. d. de, AIDAR, F. J., MATOS, D. G. de, SOUZA, R. F. de, NEVES, E. B., ARAUJO TINOCO CABRAL, B. G. de, CARMARGO, E. A., and REIS, V. M. 2020. Evaluation of Muscle Damage, Body Temperature, Peak Torque, and Fatigue Index in Three Different Methods of Strength Gain. *International Journal of Exercise Science* 13, 3, 1352–1365.

[2] Al-Nakhli, H. H., Petrofsky, J. S., Laymon, M. S., and Berk, L. S. 2012. The use of thermal infra-red imaging to detect delayed onset muscle soreness. *Journal of Visualized Experiments : JoVE,* 59.

[3] Brandenburg, J. E., Eby, S. F., Song, P., Zhao, H., Brault, J. S., Chen, S., and An, K.-N. 2014. Ultrasound Elastography: The New Frontier in Direct Measurement of Muscle Stiffness. *Archives of physical medicine and rehabilitation* 95, 11, 2207–2219.

[4] da Silva, W., Machado, Á. S., Lemos, A. L., Andrade, C. F. de, Priego-Quesada, J. I., and Carpes, F. P. 2021. Relationship between exercise-induced muscle soreness, pain thresholds, and skin temperature in men and women. *Journal of Thermal Biology* 100, 103051.

[5] Hunter, A. M., Galloway, S. D. R., Smith, I. J., Tallent, J., Ditroilo, M., Fairweather, M. M., and Howatson, G. 2012. Assessment of eccentric exercise-induced muscle damage of the elbow flexors by tensiomyography. *Journal of electromyography and kinesiology : official journal of the International Society of Electrophysiological Kinesiology* 22, 3, 334–341.

[6] Kamiş, O., Gürses, V. V., Şendur, H. N., Altunsoy, M., Pekel, H. A., Yıldırım, E., and Aydos, L. 2024. Low-Load Resistance Exercise With Blood Flow Restriction Versus High-Load Resistance Exercise on Hamstring Muscle Adaptations in Recreationally Trained Men. *Journal of Strength & Conditioning Research*.

[7] Macgregor, L. and Biancone, V. 2022. *Adaptations to Low–Load Resistance Training with Blood-Flow Restriction*.

[8] Stewart, I. B., Moghadam, P., Borg, D. N., Kung, T., Sikka, P., and Minett, G. M. 2020. Thermal Infrared Imaging Can Differentiate Skin Temperature Changes Associated With Intense Single Leg Exercise, But Not With Delayed Onset of Muscle Soreness. *Journal of Sports Science & Medicine* 19, 3, 469–477.
